# Assessment of genetic and metabolite associations of branched chain amino acids with metabolic disease in the UK Biobank using Mendelian randomization

**DOI:** 10.1186/s12920-025-02232-2

**Published:** 2025-10-16

**Authors:** Jedrzej Konarkowski, Courtney Astore, Greg Gibson

**Affiliations:** School of Biological Sciences and Center for Integrative Genomics, Georgia Institute of Technology, Atlanta, GA 30332 USA

**Keywords:** Branched-chain amino acids, Mendelian randomization, Obesity, Diabetes, UK Biobank, Metabolic disease

## Abstract

**Background:**

As the building blocks of proteins and precursors of many other important compounds, amino acids play a vital role in the biochemical processes needed to sustain life. The branched-chain amino acids (BCAAs) are unique in their structure and function, as they are metabolized in muscle tissue and play important roles in protein synthesis and energy production. However, despite their physiological importance, relatively little integrative research has been conducted into the direct relationships between this class of metabolites and their effect on risk for metabolic diseases.

**Methods:**

Utilizing an integrative PheWAS approach using UK Biobank data, we were able to identify strong, high confidence, metabolite-disease correlations for the three BCAAs: leucine, isoleucine, and valine. Relationships were established through comparison of metabolite level-disease prevalence associations with polygenic scores for BCAAs, followed by Mendelian randomization analysis.

**Results:**

All BCAAs studied demonstrated especially strong relationships with type II diabetes, and robust relationships with obesity, hypertension, sleep apnea, and chronic kidney disease. We illustrate this with a set of metabolite prevalence-disease risk plots that suggest differing potential for disease based on varying levels of branched-chain amino acid metabolites. Similar results are observed with polygenic scores for plasma BCAAs. Mendelian randomization shows positive effects of leucine and isoleucine on hypertension, and either reverse causality or no clear directional relationship for other associations, notably effects of obesity and type II diabetes on all three BCAAs, with limited or borderline evidence for other outcomes.

**Conclusions:**

Overall, the results of our study highlight a relatively unexplored area of metabolite-disease associations and provide a blueprint for uncovering additional relationships using readily available biobank data.

**Supplementary Information:**

The online version contains supplementary material available at 10.1186/s12920-025-02232-2.

##  Background

Amino acids play a vital role in metabolism, serving not only as the foundations of protein synthesis but also as crucial components of cell growth, metabolism, immunity, and signaling pathways. Out of the twenty different proteinogenic amino acids, leucine (Leu), isoleucine (Ile), and valine (Val) are considered branched-chain amino acids (BCAAs). BCAAs are defined by their distinct molecular structure, namely branched sidechains as opposed to the linear sidechains of other amino acids. BCAAs in humans are sourced from the diet or gut microbial biosynthesis and catabolizes uniquely by a pathway which bypasses the liver, where other amino acids are catabolized, instead occurring in skeletal muscle and other peripheral tissues through the activity of the branched-chain amino acid aminotransferase (BCAT1/BCAT2) enzymes [1].

Due to their involvement in a multitude of important metabolic pathways and ability to be quantified in blood testing, several correlational associations between BCAA levels and disease have been identified, including elevated BCAA levels in maple syrup urine disease (MSUD) [2], type II diabetes [3–7], and obesity [8]. Further research has identified low levels of BCAAs to be associated with chronic kidney disease [9–11] liver cirrhosis [12], and urea cycle disorders [13]. While these studies provide a useful foundation for understanding the roles BCAAs play in pathogenesis and disease risk, they do not establish any causal relationships and may be influenced by confounding variables. Genome-wide association studies (GWAS) have been an important next step toward developing a greater understanding of causality, highlighting relevant regions of the genome and identifying specific, single-nucleotide genetic markers of disease called single-nucleotide polymorphisms (SNPs). To date, GWAS has been conducted on a variety of phenotypes, including diseases associated with BCAAs through previous research such as chronic kidney disease (CKD) [14], type II diabetes (T2D) [15], hypertension [16], obesity [17], and sleep apnea (SA) [18]. GWAS has also been conducted on metabolic biomarkers including amino acids [19, 20], providing additional insights into the relationships between metabolites, genetics, and disease. Until recently, the few human association studies had low sample sizes, but the recent publication of a large-scale GWAS for over 200 different circulating metabolites [21] has opened new opportunities for analyzing the genetic and metabolic correlates of disease.

GWAS data has led to the development of several new statistical approaches for understanding the relationships between genotypes and phenotypes. Many recent studies have employed Mendelian randomization (MR), a statistical method that treats genetic variants as natural experiments to investigate whether a specific factor causally affects disease status [22]. This approach is based on the principle that genetic variants are randomly assigned at conception and are therefore not influenced by lifestyle or environmental factors, reducing confounding and reverse causation, and providing a plausible inference of causality [23]. MR studies have investigated the relationship between BCAAs and several diseases. Causal effects of BCAA levels have been inferred with variable strength of evidence in cardiovascular disease [22, 24–27], hypertension [28, 29], Alzheimer’s disease [30, 31], specific cancers [32–34], type II diabetes [35, 36], and obesity [26]. Despite evidence of causal relationships between BCAAs and various disorders, the limited number of studies to date and inconsistencies in findings for the same disorder have made it difficult to confidently establish causality or rank the relative strength of these relationships. Much recent progress in this domain has been possible through the increased sample sizes available through large-scale biobank analysis, particularly utilizing integrative PheWAS methods to analyze metabolite-disease associations [37, 38]. Our primary objective was to further demonstrate the utility of integrative PheWAS using biobank data to identify and explore metabolic-disease associations, and to characterize the specific genetic contributions to these relationships through polygenic scores and MR. For our study, the UK Biobank [39] proved a transformative resource for studying metabolite-disease associations, providing access to detailed metabolomic and genetic information from over 500,000 participants. Additionally, the longitudinal nature of the dataset, combined with detailed lifestyle, environmental, and medical records, allowed us to better account for confounding variables and explore causal links between metabolites and disease outcomes.

##  Methods

### Dataset

Our analysis utilized the UK Biobank (UKB) cohort [39] consisting of 502,364 individuals in the United Kingdom, performed under project approval number 17,984. Both genotypic and phenotypic data were utilized.

Following data filtering and quality control, data associated with 152,199 unique individuals was used in the final study, namely 30.3% of the total sample. The exclusion criteria were violation of the following: kinship found (ID: 22021; ~150,000 excluded), White British only (ID: 22006; n = 409,551), self-reported sex matches genetic sex (ID: 22001, 31), no sex chromosome aneuploidy (ID: 22019), no information present for age (ID: 21022), no information present for sex (ID:31), and no information present for one or more of the covariates (ID:22009). A further approximately 130,000 individuals were excluded because there was no available metabolite data for leucine (ID: 23466), isoleucine (ID:23465), or valine (ID: 23467). The primary phenotypic data utilized in our analyses consisted of the aforementioned BCAA levels as well as disease case/control status.

The genotypic component of our study included the calculation of polygenic scores (PGS) for leucine, isoleucine, and valine using data sourced from a 2024 consortium study covering approximately 12 million SNPs in over 135,000 individuals who did not overlap with the UK Biobank, instead relying on 33 cohorts primarily from continental Europe (120,241 individuals/88.4% of total), China (4435 individuals/3.3%), and South Asia (11,340 individuals/8.3%) [21].

### Phenotypic associations

Although the 250 metabolite levels generated on the targeted NMR panel by Nightingale Health are reported in mmol/L, we elected conservatively to normalize the BCAA data prior to any analysis using an R script. This was done using z-scores calculated for individuals’ metabolite levels using the standard formula z = (x-µ)/σ, where x is the metabolite level, µ is the mean of that metabolite across the entire sample, and σ is the standard deviation. In the case that an individual had multiple reported values of a metabolite due to data collection at two separate times, the median of the two values was utilized in the z-score calculation. Correct calculation of z-scores was visually verified by evaluating the distributions via generation of histograms and summary statistics for the metabolite levels before and after normalization and removal of outliers four standard deviations from the mean.

Next, logistic regression was performed utilizing another R script to evaluate the associations between the aforementioned metabolite level z-scores and prevalent case/control status for 1755 ICD-10 disease-based phecodes (Supplementary Table 1) [40] in the UKB dataset. However, diseases which had fewer than 50 cases in the dataset were automatically excluded, leaving 1094 Phecodes in the final analysis. The covariates included in the regression model were age, sex, and the first 10 genetic principal components. The log-odds effect estimate, standard error, z-score of effect size, upper and lower confidence intervals, odds ratio, and p-value were calculated (Supplementary Table 2). Significance of associations was based on a p-value threshold of 10^−5^, based on 0.05 divided by the product of the total number of diseases in the dataset (1755) and the number of metabolites being tested [3].

###  Genotypic associations

Polygenic scores (PGS) were calculated for leucine, isoleucine, and valine using data sourced from the NHGRI-EBI GWAS Catalog [21] from a non-UK Biobank largely European ancestry cohort. First, the GWAS summary statistics were reformatted using a Python script in preparation for use with PRS-CS v1.1.0. Polygenic Risk Score - Continuous Shrinkage (PRS-CS) [41] is a Bayesian regression framework used for PGS computation which integrates GWAS summary statistics and linkage disequilibrium (LD) patterns, applying a continuous shrinkage prior to improve prediction accuracy. SNP effect sizes were retained with their signs, such that both positive and negative associations contributed directionally to the final score. The estimated effect sizes from PRS-CS (Supplementary Table 3) were subsequently used in PGS generation using PLINK 2.0 alpha 5.25 [42].

The PLINK 2.0 outputs were summed across all chromosomes to generate a PGS value for each individual for leucine, isoleucine, and valine. Then, logistic regressions for predicting disease status were computed, utilizing the same covariates as in the phenotypic analysis but with PGS percentile instead of metabolite level z-score. For consistency we chose to use the same covariates for all diseases but acknowledge that additional adjustments for measures of obesity or smoking status, for example, may alter some associations. To assess shared genetic architecture between the three BCAAs, we also computed Pearson correlation coefficients between the three PGS computed in the UK Biobank cohort participants included in our analyses. While not formal genetic correlations (r_g_), these provide a similarly valuable indication of the degree of shared genetic influences.

###  Bidirectional Mendelian randomization

In order to determine the directionality and potential causality of the metabolite-disease relationship, we conducted bidirectional Mendelian randomization studies on each metabolite-disease pair. The first part of the analysis utilized genetic variants that associate with BCAA levels as instruments to investigate a potential causal effect on increased risk of metabolic disease, while the second part examined the reverse relationship, employing SNPs for a given disease as instrumental variables to examine whether the disease has a causal influence on BCAA levels. In both cases the instruments are chosen for genome-wide significance, and the analysis is essentially a test of whether their effect sizes on the outcome are correlated with effect sizes of the same SNPs for the exposure on the outcome regardless of significance. In each analysis, a metabolite or disease trait was treated as the exposure and the other as the outcome. GWAS summary statistics for BCAAs were sourced from the aforementioned consortium study which identified variants associated with the 233 different circulating metabolites present in the UK Biobank [21]. The 136,016 participants came from 33 cohorts and were primarily from continental Europe (120,241 individuals/88.4% of total), although there were also individuals from China (4435 individuals/3.3%) and South Asia (11,340 individuals/8.3%). Study accession GCST90301994 was used for leucine, GCST90301987 for isoleucine, and GCST90302122 for valine.

GWAS summary statistics for diseases were sourced from several different publications. Type II diabetes GWAS data was sourced from the European-ancestry dataset from Mahjan and the DIAMANTE consortium [43], with a sample of 933,970 individuals from 32 different cohorts, of which 80,154 were cases and 853,816 were controls based on ICD codes for T2D or T2D complications in participant health records or purchases of T2D treatment drugs. Sleep apnea (SA) GWAS data was sourced from Campos et al. [44] and consisted of 362,638 European-ancestry individuals from five different datasets: UK Biobank, the Canadian Longitudinal Study of Aging, the Australian Genetics of Depression Study, Partners Healthcare Biobank, and FinnGen. There were 25,008 cases and 337,630 controls, and disease status was either self-reported or sourced from ICD codes in electronic health records. Notably, some SNPs used as genetic instruments for the BCAAs were not available in this SA GWAS data and therefore did not contribute to the SA MR analyses.

In some cases, studies of continuous traits closely linked to the disease were used instead of the disease itself due to greater statistical power resulting from a larger sample size. The systolic blood pressure (SBP) GWAS from Keaton et al. [45] was utilized for hypertension due to its sample size of 1,028,980, the largest of any such study to date. The participants were all of European ancestry and were sourced from UK Biobank, the International Consortium for Blood Pressure, Million Veteran Program and BioVU. The Stanzick et al. GWAS of estimated glomerular filtration rate (eGFR) [46] was used for CKD, specifically the EUR dataset consisting of 1,004,040 European-ancestry participants sourced from the CKDGen Consortium and UK Biobank. The body mass index (BMI) GWAS from Pulit et al. [47] was utilized for obesity, consisting of 694,649 European-ancestry participants from the GIANT Consortium and UK Biobank.

The interpretation of MR effect estimates depends on the units of the exposure and outcome variable from the source GWAS. For binary disease traits including T2D and SA, the estimates are reported on a log-odds scale. For the continuous traits, there is more variation, with BCAAs and BMI being inverse rank normal transformed, so the units are in standard deviations on the rank-normal (z) scale. eGFR was natural-log transformed, so the units are ln(mL/min/1.73 m²). SBP was not transformed at all, so its units are in mmHg.

Our MR analyses rely on three core instrumental variable assumptions. The first assumption is relevance, which assumes that genetic variants are robustly associated with the exposure. The second is independence, which assumes that there are no confounders of the instrument-outcome relationship. The final assumption is exclusion restriction, that instruments only influence the outcome through the exposure.

Prior to conducting any analyses, each dataset was cleaned and reformatted to a common column standard (SNP, effect_allele, other_allele, beta, se, pval, eaf) to ensure compatibility with downstream data processing scripts. For the exposure data, we selected the genetic variants associated with the trait at genome-wide significance (p < 5 × 10⁻⁸) and applied linkage disequilibrium (LD) pruning (r² < 0.001 within a 10 Mb window) using 1000Genomes EUR LD blocks [48] and PLINK 1.9 beta 7.7 [49] to ensure independence of instruments for MR. For the outcome data, we retained all SNPs to maximize overlap during harmonization. We performed all MR in R 4.5.0 using a custom script utilizing the TwoSampleMR package v0.6.15 [50]. First, exposure and outcome datasets were harmonized to correct for strand inconsistencies and ensure the effect of a SNP on the exposure and outcome corresponds to the same allele, accounting for any inversions. The harmonization process also removed missing data as well as ambiguous or palindromic SNPs with an estimated allele frequency (EAF) between 0.42 and 0.58. Then, MR was conducted using inverse variance weighted (IVW) as the primary analysis for association discovery. Simple mode, weighted mode, MR-Egger and weighted median were conducted as well to assess the robustness of the analysis under weaker assumptions. As we utilized GWAS data that had been adjusted for covariates during primary association testing, no further adjustment was conducted along our MR pipeline.

##  Results

### Comprehensive PheWAS

**Fig. 1:**
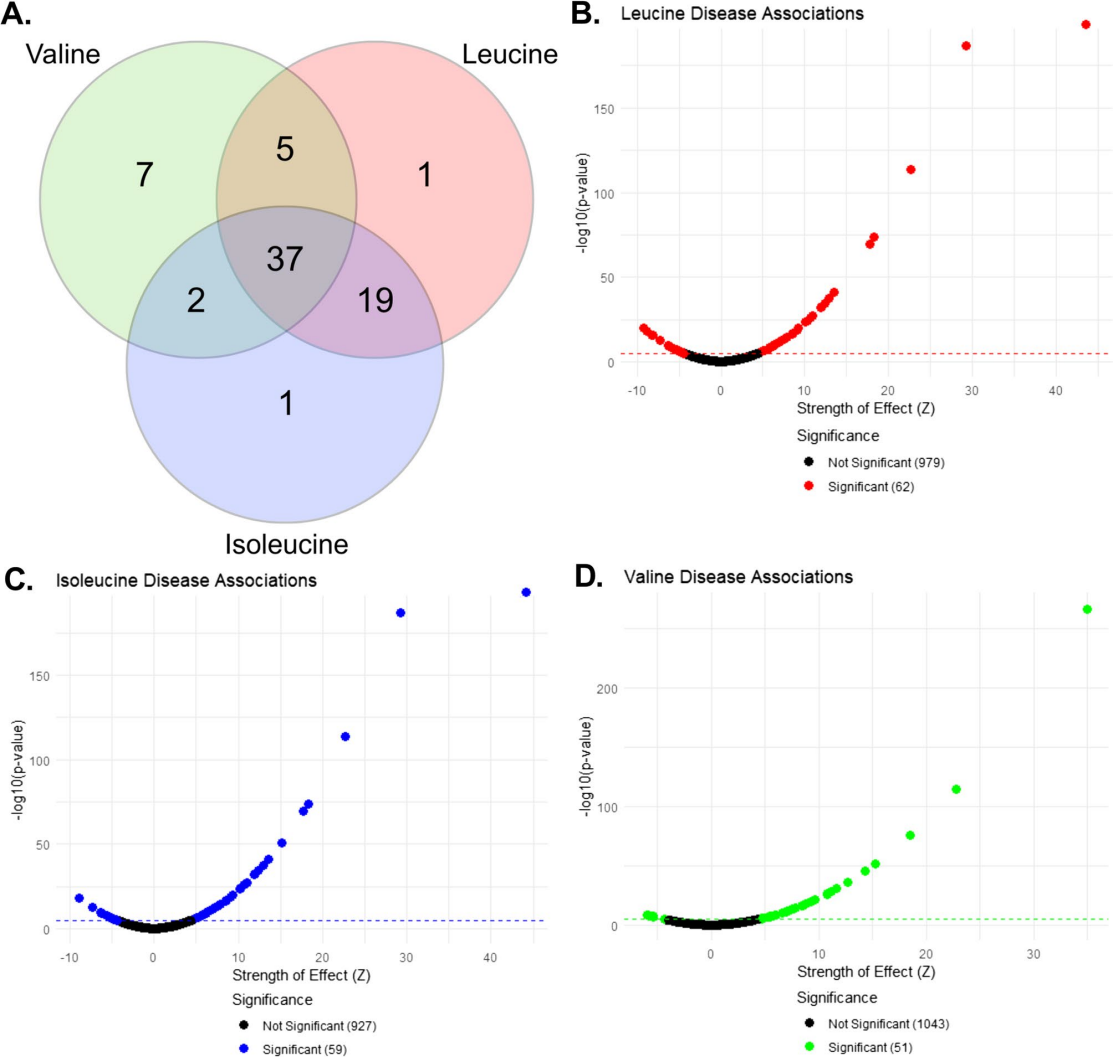
**A** Venn diagram of overlapping disease associations between leucine, isoleucine, and valine. **B**, **C**, **D** Individual disease associations for leucine, isoleucine, and valine, respectively, plotted against their strength of effect (y-axis). Strength of effect is a z-score, calculated as the log-odds ratio (beta) divided by standard error

Initial PheWAS analysis identified disease associations with p-values ranging from the Bonferroni-adjusted significance threshold of 1 × 10^−5^ to around 1 × 10^−187^for the associations with the strongest evidence against the null hypothesis. The highest number of associations overall was identified with leucine with a total of 62, followed by isoleucine at 59 and valine at 51 (Fig. 1A). The effect sizes (β) and corresponding standard errors (SE) for these associations ranged from β = −0.38 to 0.58 (SE: 0.0068–0.0894) for leucine, β = −0.38 to 0.58 (SE: 0.0068–0.0894) for isoleucine, and β = −0.27 to 0.53 (SE: 0.0069–0.0892) for valine. There was a high degree of similarity between leucine and isoleucine in disease associations, with 19 associations shared between the two and not shared with valine. For all three BCAAs, the vast majority of associations indicate a positive relationship where higher circulating metabolite levels correspond to higher prevalence of disease (40/62 for Leu, 40/59 for Ile, and 45/51 for Val; Fig. 1B, C, D). A full listing and visualization of metabolite-disease associations is provided in Supplementary Tables 2 and Supplementary Figure 1.

### Specific metabolite-disease associations

**Fig. 2:**
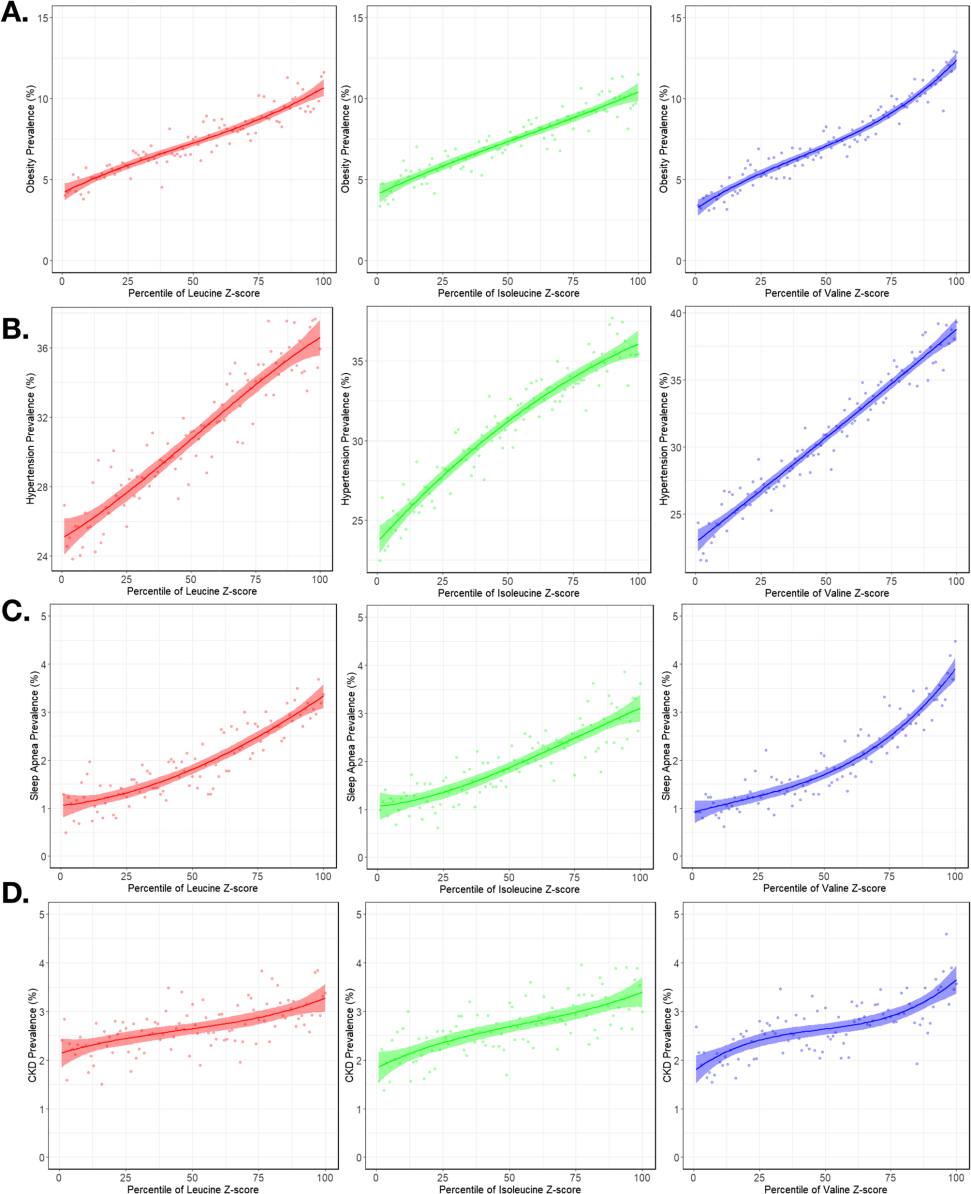
Metabolite level-disease prevalence curves for the observational relationship between BCAA z-scores (x-axis) and prevalence (y-axis) of obesity **A**, hypertension **B**, sleep apnea **C**, and chronic kidney disease **D**. Each dot represents the observed disease prevalence within a single percentile of the metabolite level distribution. The curve is a cubic polynomial regression fit to these points

To better visualize the relationships between metabolite levels and disease prevalence, we plotted prevalence against the percentile of metabolite z-scores (Fig. 2). The beta coefficients (β) from our logistic regression models represent the change in the log-odds of a disease per one standard deviation (1-SD) increase in the standardized BCAA level. The strongest evidence of association was consistently observed with type II diabetes. For this analysis, which included 9771 cases and 117,175 controls for leucine, a 1-SD increase was associated with a 57% increase in the odds of having type II diabetes (Odds Ratio [OR] = 1.57, 95% CI: 1.54–1.60; β = 0.45, SE = 0.01, *p* < 1 × 10⁻³⁰⁰). The effects for isoleucine (β = 0.45, SE = 0.01, p < 1 × 10⁻³⁰⁰; 9771 cases, 117,176 controls) and valine (β = 0.36, SE = 0.01, p < 1 × 10⁻³⁰⁰; 9759 cases, 117,023 controls) were similarly large. Following diabetes, the next largest effect sizes were seen with obesity. For leucine, the analysis of 9387 cases and 118,515 controls yielded a beta of 0.31 (SE = 0.01), corresponding to a 36% increase in odds per 1-SD increase in leucine levels. The associations for isoleucine (β = 0.31, SE = 0.01) and valine (β = 0.24, SE = 0.01) were also notable. Strong evidence was also found for an association with sleep apnea. The effect sizes for leucine (β = 0.27, SE = 0.02; 2448 cases), isoleucine (β = 0.27, SE = 0.02; 2449 cases), and valine (β = 0.22, SE = 0.02; 2442 cases) were all substantial. Finally, more modest but clear relationships were observed for chronic kidney disease, with betas for leucine, isoleucine, and valine of 0.14 (SE = 0.02), 0.14 (SE = 0.02), and 0.14 (SE = 0.02), respectively, and hypertension, with betas of 0.15 (SE = 0.01), 0.15 (SE = 0.01), and 0.13 (SE = 0.01). Note that the slight differences in case and control numbers for each are due to missing values for specific BCAAs in some individuals.

While there was variance in the absolute prevalence of disease in the UKB dataset, with hypertension having the highest prevalence, followed by obesity, CKD, and sleep apnea, the results clearly indicate that higher levels of BCAA are associated with higher disease risk in all four cases (Fig. 2).

### Metabolite PRS-disease associations

**Fig. 3:**
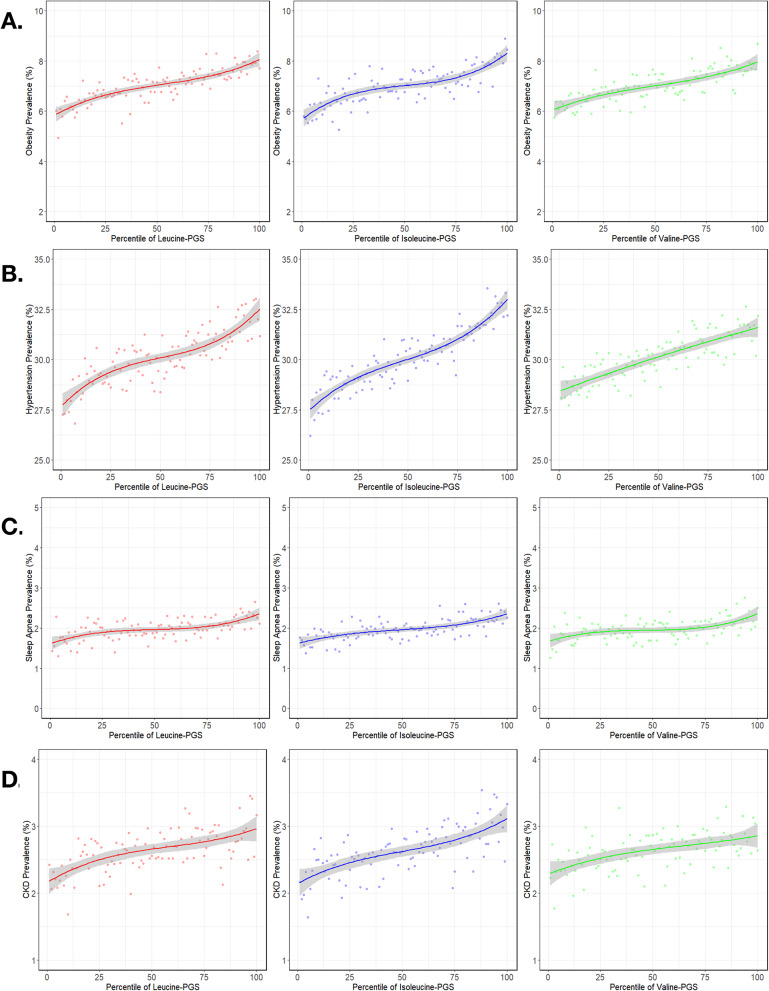
Metabolite PGS-disease prevalence curves for the relationship between BCAA PGS (x-axis) and prevalence (y-axis) of obesity **A**, hypertension **B**, sleep apnea** C**, and chronic kidney disease **D**. Dots represent prevalence of the disease at each individual percentile of PGS for the respective metabolite PGS. Curves were derived from a cubic polynomial regression of disease prevalence on metabolite PGS

The PGS for each BCAA shows a similar relationship to disease prevalence as the metabolite level itself. As expected, there is also a high degree of correlation across PGS for the three BCAAs, consistent with shared genetic architecture, ranging from 0.79 for valine-isoleucine to 0.85 valine-leucine and 0.91 for leucine-isoleucine. The panels in Fig. 3 show that in each case the PGS captures less of the variance than the actual metabolite levels (as expected since SNP heritabilities are less than 30%). They all follow the characteristic inverted S-shape characteristic of prevalence-PGS percentile relationships due to the normal distribution of underlying scores. However, this is in many cases attenuated as the inflections at the extremes are less than typically observed for disease PGS, namely 3 to 5-fold differences in prevalence between the lowest and highest deciles [51]. For example, considering hypertension (Fig. 3B), individuals in the top decile of polygenic score for either leucine or isoleucine are just 15% more likely to have the disease than those in the bottom decile. Despite differences in overall prevalence, the comparable differences are 25% for obesity and sleep apnea (Fig. 3A and C, respectively), and 30% for chronic kidney disease (Fig. 3D).

### Bidirectional Mendelian randomization

**Fig. 4:**
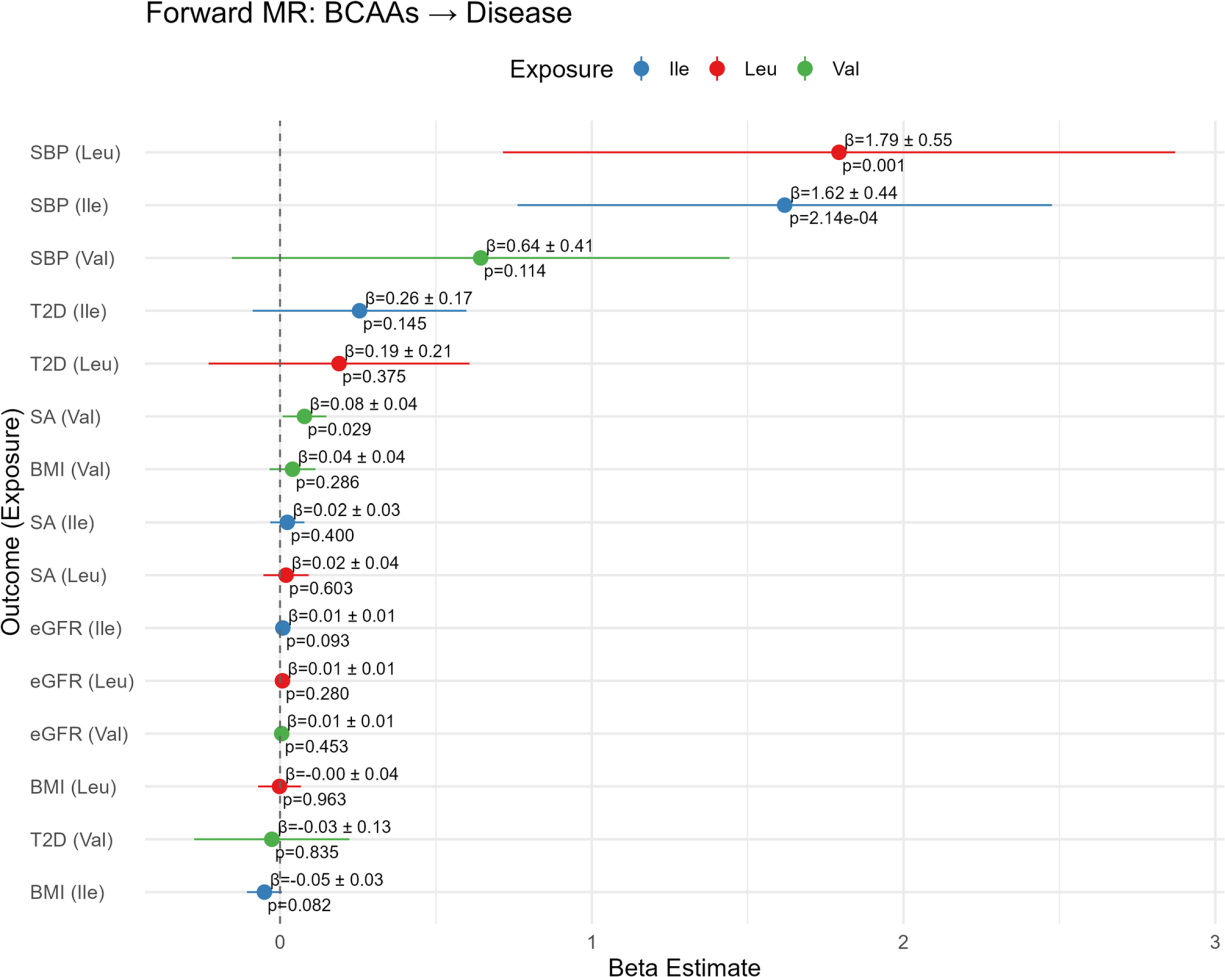
Mendelian randomization IVW forest plot of the forward direction with BCAAs as the exposure and diseases as the outcomes. Beta, standard error, and p-value are provided alongside a visual representation of beta on a linear scale. The beta estimate represents the change in the disease outcome per one rank normal standard deviation (SD) unit increase in the BCAA exposure. For binary outcomes (T2D, SA), a one-unit increase corresponds to a one-unit increase in the log-odds of having the disease; For SBP a one-unit increase corresponds to a 1mmHg increase. For BMI a one-unit increase corresponds to a 1 SD increase on the inverse-rank normal (z) scale. For eGFR, a one-unit increase corresponds to a one-unit increase on the natural log scale of mL/min/1.73 m²

In order to evaluate whether the prevalence-risk relationships reported above might reflect causal impacts of metabolite levels on disease prevalence, we next performed bi-directional Mendelian randomization. The instruments used in our analysis are listed in Supplementary Table 4. In the forward MR direction, where BCAAs were the exposure and diseases were the outcome, most associations showed either no or weak evidence of causality (Fig. 4), with the exception of isoleucine with SBP (β = 1.62; SE: 0.44; p = 2.14 × 10⁻⁴) and leucine with SBP (β = 1.79; SE: 0.55; p = 0.001), both of which showed positive associations. A much weaker effect was observed between valine and SBP (β = 0.64; SE: 0.41; p = 0.114).

**Fig. 5:**
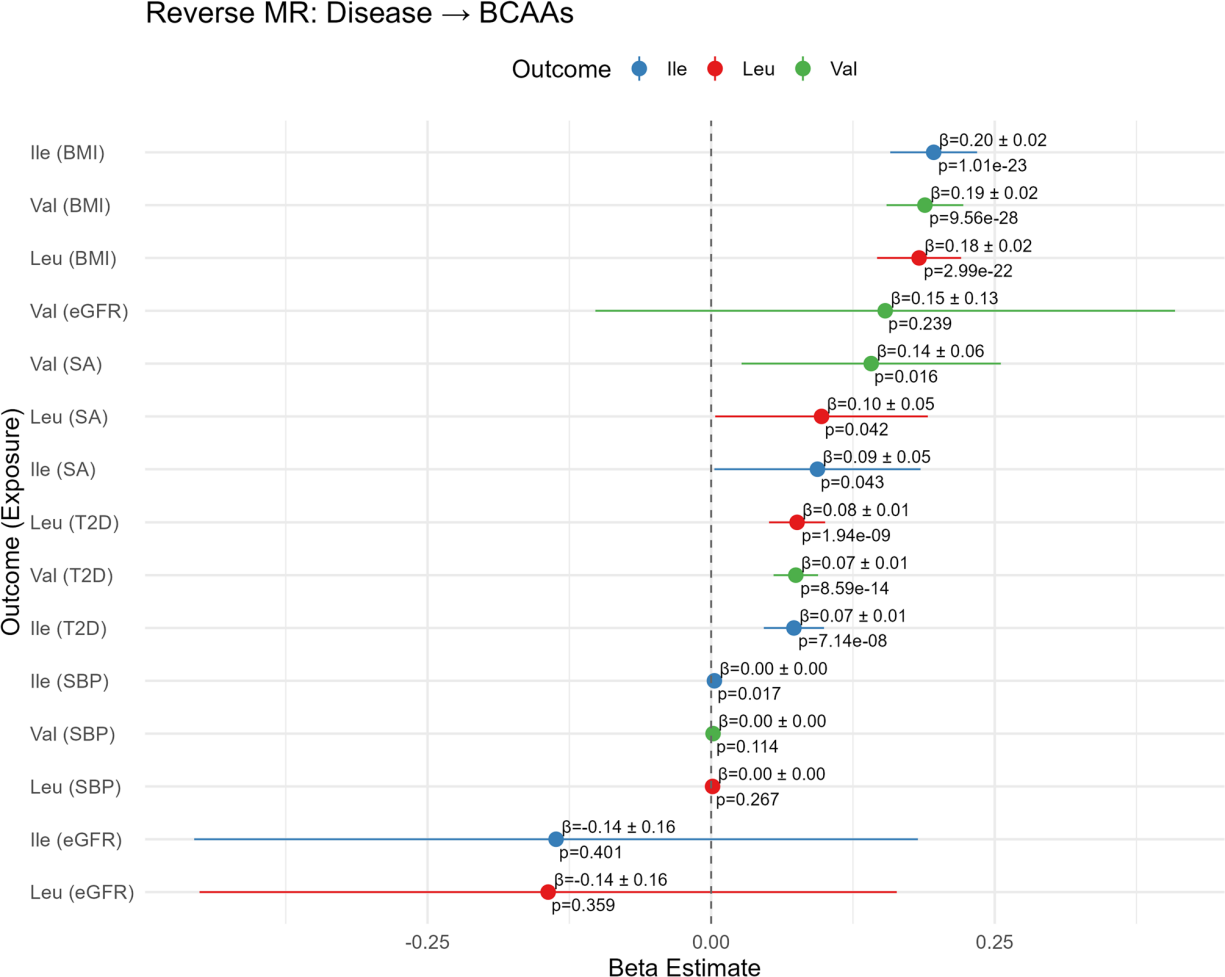
Mendelian randomization IVW forest plot of the reverse direction with diseases as the exposure and BCAAs as the outcomes. Beta, standard error, and p-value are provided alongside a visual representation of beta on a linear scale. The beta estimate represents the change in BCAA levels (in rank normal standard deviation units) per one-unit increase in the exposure. For binary outcomes (T2D, SA), a one-unit increase corresponds to a one-unit increase in the log-odds of having the disease; For SBP a one-unit increase corresponds to a 1mmHg increase. For BMI a one-unit increase corresponds to a 1 SD increase on the inverse rank-normal (z) scale. For eGFR, a one-unit increase corresponds to a one-unit increase on the natural log scale of mL/min/1.73 m²

In the reverse direction, with disease as the exposure and BCAAs as the outcome, stronger and more consistent effects were observed (Fig. 5). The strongest relationship appears to be BMI on all three BCAAs including valine (β = 0.19; SE: 0.02;p = 9.56 × 10⁻^28^), isoleucine (β = 0.20; SE: 0.02; p = 1.01 × 10⁻^23^), and leucine (β = 0.18; SE: 0.02; p = 2.99 × 10⁻^22^). There is also strong evidence for an effect of type 2 diabetes (T2D) liability on all three BCAAs (see next section), including valine (β = 0.07; SE: 0.01; p = 8.59 × 10⁻¹⁴), isoleucine (β = 0.07; SE: 0.01; p = 7.14 × 10⁻⁸), and leucine (β = 0.08; SE: 0.01; p = 1.94 × 10⁻⁹). We also found suggestive evidence for associations of sleep apnea liability with valine levels (β = 0.14; SE: 0.06; p = 0.016). In these analyses, the beta coefficient (β) represents the standard deviation (SD) change in BCAA levels per one-unit increase in the log-odds of the genetically predicted disease, interpreted as a life-course effect of disease liability. All estimates and p-values were derived from inverse variance weighted (IVW) Mendelian randomization analyses. Additional methods such as simple mode and weighted mode as well as sensitivity analyses (MR-Egger, weighted median) are reported in Supplementary Tables 5 and Supplementary Figs. 2–6 with results on the individual SNP level in Supplementary Tables 6 and are generally consistent with the main findings. MR-Egger intercept tests did not indicate significant directional pleiotropy for most metabolite-disease pairs (all p > 0.05), apart from Ile with eGFR (p = 2.9 × 10⁻⁴), Leu with eGFR (p = 0.0096), and T2D with Val (p = 0.017) relationships.

We also performed Cochran’s Q tests to assess heterogeneity and MR-PRESSO to identify and correct outlier instruments in our initial analysis, and these additional analyses are reported in Supplementary Tables 7 and 8 respectively. We found that several pairings demonstrated very high heterogeneity, including BMI with Isoleucine (Q_IVW = 1315.34), BMI with Leucine (Q_IVW = 1243.52), BMI with Valine (Q_IVW = 1022.68), the Ile with SBP pairing (Q_IVW = 428.65), Leu with SBP (Q_IVW = 377.32), and Ile with T2D (Q_IVW = 945.04). In the reverse direction, pairings such as Ile with eGFR (Q_IVW = 491.15; MR-PRESSO β_raw = 0.011; β_corrected = 0.0054) and Leu with BMI (Q_IVW = 323.42; MR-PRESSO β_raw = − 0.012; β_corrected = 0.049) exhibited both heterogeneity and major changes in effect size following outlier removal, further supporting the presence of pleiotropic distortion. Therefore, these associations should be interpreted with caution.

### Diabetes

**Fig. 6:**
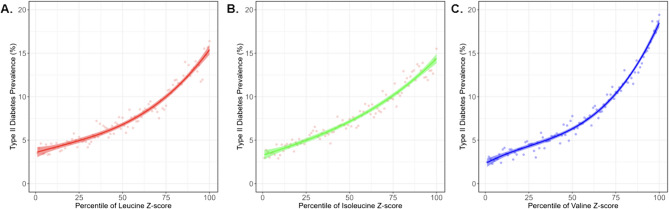
Metabolite-disease plot for (A) leucine, (B) isoleucine, and (C) valine and type II diabetes

For type II diabetes, all three BCAA show strong relationships between metabolite levels and disease prevalence (Fig. 6). Valine metabolite levels generated the greatest discrimination between the top and bottom deciles of risk, namely 17% versus 3%, a 6-fold difference (compared with up to 4-fold for the leucine and isoleucine). Notably, associations with subtypes of diabetes with particular clinical manifestations (ophthalmic, neurological, peripheral circulatory, and renal) were also very strong (Supplementary Fig. 1).

**Fig. 7:**
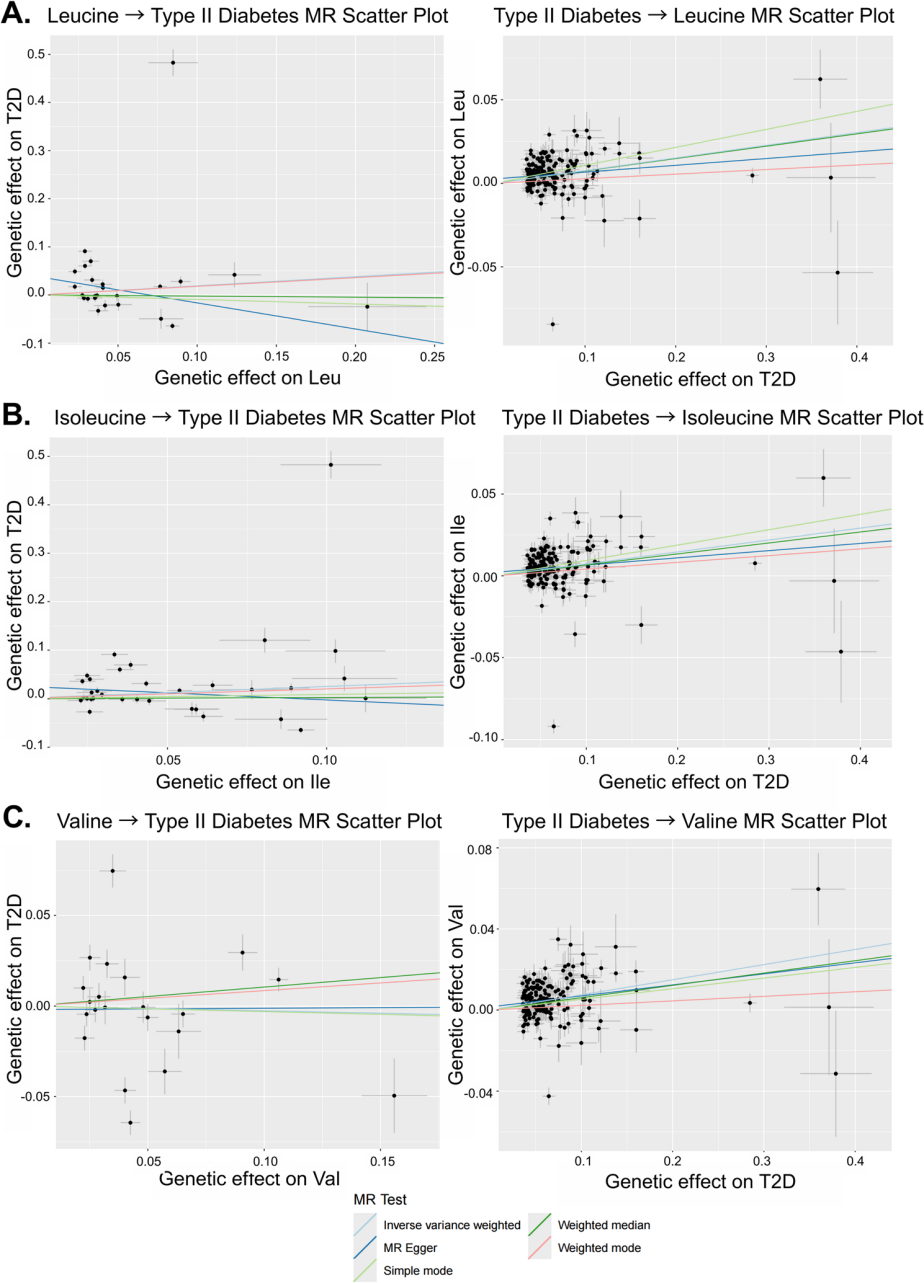
Bidirectional Mendelian randomization scatter plots for ** A **leucine, **B** isoleucine, and **C** valine and type II diabetes

However, the relationship between BCAAs and type II diabetes is for the most part one of reverse-causation, since there is a robust causal effect of T2D on all three BCAAs (Fig. 5). Closer examination of the relationship using MR scatter plots in Fig. 7 shows that positive slopes are visible across all three BCAAs in IVW as well as the other MR methods, indicating robustness of the associations. However, there is considerable heterogeneity among the tests, visible in Fig. 7 as variable slopes, and confirmed with high Cochran’s Q values (Supplementary Table 7). Conversely, the BCAA to T2D panels on the left show relatively flat trendlines and high relative dispersion of instruments, consistent with the lack of a causal relationship of BCAAs on T2D observed previously. A possible exception is isoleucine which has the largest slope of the IV regression, and we also note that both weighted modes provide some evidence that valine may influence diabetes risk. However, the association from T2D to valine shows a statistically significant MR-Egger intercept (p = 0.017), indicating potential directional horizontal pleiotropy in this relationship. This finding, combined with high heterogeneity makes the T2D to valine causal estimate less reliable than those for leucine and isoleucine. MR-PRESSO supports the robustness of the broader reverse causal relationship, with outlier-corrected beta values being consistently higher compared to the initial estimate across Ile (β_raw = 0.072; β_corrected = 0.083), Leu (β_raw = 0.074; β_corrected = 0.083), and Val (β_raw = 0.072; β_corrected = 0.074).

##  Discussion

Overall, our analyses identified over 50 strong associations between BCAA levels and a wide range of diseases, notably involving positive relationships for all three BCAAs with type II diabetes, hypertension, obesity, sleep apnea, and chronic kidney disease (CKD). Further analysis of each individual metabolite-disease relationship showed that across all diseases and all BCAAs, higher levels of each amino acid in the blood are most often correlated with higher risk of disease. Notably, the magnitude of the effect varied between diseases, with a smaller difference in CKD rates compared to the other four major diseases. As expected, the comparison of disease rates against PRS for each BCAA shows a very similar pattern, demonstrating the efficacy of the PRS in predicting metabolite levels despite the lower variance explained. Valine PRS showed the strongest influence on type II diabetes, while isoleucine and leucine were stronger for the other diseases, showing that the predictions are not simply a function of the heritability explained by the scores. Although the major sources of BCAAs in the body are from our diets [52] and our microbiomes [53], the associations with PGS show that genetic influences on BCAA metabolism and catabolism contribute to disease risk.

While our observational and PGS analyses suggested strong associations between BCAAs and metabolic diseases and confirmed that genetics plays a role in these relationships, MR provides further clarification by attempting to disentangle correlation from causation. Broadly, our MR results present a more complex picture, namely that while BCAAs like leucine and isoleucine have some causal effect on blood pressure and hypertension, other diseases such as type II diabetes or obesity (as measured by BMI over 30) show a reverse causal effect where disease modulates BCAA levels while BCAA levels have little effect on causing the disease. Our analyses also show limited evidence for positive causality of sleep apnea on valine levels or isoleucine on hypertension. However, the robustness of nearly all causal relationships is challenged by high heterogeneity, implying widespread horizontal pleiotropy. While MR-Egger suggests a lack of directional pleiotropy for most findings, the heterogeneity indicated by our other sensitivity analyses suggests the true causal effect size may differ from our estimates.

Even so, our strongest effect size findings are consistent with the literature - Würtz et al. [54] previously demonstrated in a large Finnish cohort that genetic predisposition to BMI mediates both SBP and BCAA, and here we show that BMI strongly mediates BCAAs while BCAAs mediate hypertension. Therefore, while our analysis shows some clear directional effects, such as T2D and BMI causally elevating BCAAs, and a suggestive reverse effect from BCAAs to SBP, these relationships are likely complex and mediated by numerous biological pathways. Overall, our MR clearly demonstrates how positive associations between a metabolite level or metabolite PGS and disease prevalence may not imply causation, rather simply reflecting how genetic associations with the metabolite may be confounded by the influence of disease in actually increasing the BCAA. Multivariate MR might be used in future studies to explore such dependencies.

Interestingly, outside of the relationship between BCAAs and type II diabetes, our PheWAS shows there are also strong relationships with other specific subtypes of type II diabetes, namely type II diabetes with ophthalmic, neurological, peripheral circulatory, and renal manifestations (Supplementary Fig. 1). The ophthalmic manifestations are likely closely tied with diabetic retinopathy, which results from the adverse effects of elevated blood pressure in blood vessels in the eye. The role of BCAAs in these relationships is not fully understood, though a potential explanation could involve the ability of BCAAs to induce insulin release, which leads to an increase in overall blood pressure [28]. The peripheral circulatory manifestations are likely linked to this causal mechanism as well, and this explanation would be consistent with our MR results. It is possible that similar biological mechanisms may contribute to the renal manifestations. Alternatively, elevated BCAAs might contribute more directly to renal issues by promoting inflammation, oxidative stress, or mitochondrial dysfunction, processes commonly linked to diabetic nephropathy. The latter explanation is supported by previous animal testing, which found increased CKD progression in rats in an amino-acid rich diet [55], though these findings are not fully generalizable to CKD in humans and our MR does not support this explanation.

Through the identification and further analysis of metabolite-disease relationships, we build on previous work [37] demonstrating the value of integrative PheWAS as a tool for better understanding how metabolites influence disease. Our work not only identifies chronic kidney disease (CKD), obesity, hypertension, and sleep apnea as diseases of interest related to BCAAs but also highlights the potential for PheWAS to uncover novel and specific associations, such as the link between BCAAs and type II diabetes with renal manifestations. By pinpointing this relationship, we underscore the ability of large-scale PheWAS to provide nuanced insights into how metabolic pathways contribute to disease subtypes, which may otherwise remain obscured in broader analyses. These findings illustrate the power of PheWAS techniques as a powerful tool for generating hypotheses for future functional studies, guiding biomarker discovery, and identifying potential therapeutic targets for complex disease.

A major limitation of our study is that our conclusions are not necessarily transferable to other population groups due to a lack of inclusion of non-European ancestry individuals in our analyses, owing to low sample size and efforts to avoid potential population stratification. This limitation underscores the need for future research to prioritize diverse cohorts to ensure findings are broadly applicable and to uncover genetic and metabolic factors that may vary across ancestries. Expanding the representation of non-European populations in genomic and metabolomic studies could enhance our understanding of how genetic and environmental factors, including diet, interact in different populations, ultimately improving the equity and efficacy of precision medicine initiatives.

We must also acknowledge that we excluded several hundred diseases from our analyses through the 50-case minimum threshold in order to maintain statistical power, and ultimately only focused on five core diseases in most of our analyses. As a result, there are hundreds of diseases, including many rare diseases, with which metabolite-disease relationships remain relatively unexplored. Furthermore, while age was included as a covariate in our PheWAS, we did not stratify our analyses between age groups. It is possible that the relationships between BCAAs and metabolic diseases vary across age groups, and future studies could explore the role of age in the relationship between metabolites and diseases in greater detail. Finally, it would be interesting to explore the relationship between polygenic scores for metabolites and incident disease, to ascertain to what degree elevated BCAAs stratify risk for progression from pre-clinical to clinical conditions.

##  Conclusions

Our study identifies over 50 highly significant disease associations with the branched-chain amino acids leucine, isoleucine, and valine and further explores the relationships of BCAAs with type II diabetes, obesity, hypertension, sleep apnea, and chronic kidney disease. Higher levels of circulating BCAAs and higher PGS for each metabolite correlate with higher disease prevalence. Bidirectional Mendelian randomization demonstrates different degrees of causal relationships in different metabolite-disease pairs. There is strong support for a positive effect of type II diabetes and obesity on BCAAs and of leucine and isoleucine on hypertension, as well as modest support for a positive causal influence of sleep apnea on valine and of isoleucine on hypertension, while all other relationships tested show little to no statistical significance.

## Supplementary Information

Supplementary Material 1.

Supplementary Material 2.

Supplementary Material 3.

Supplementary Material 4.

Supplementary Material 5.

Supplementary Material 6.

Supplementary Material 7.

Supplementary Material 8.

Supplementary Material 9.

Supplementary Material 10.

Supplementary Material 11.

Supplementary Material 12.

Supplementary Material 13.

Supplementary Material 14.

Supplementary Material 15.

Supplementary Material 16.

Supplementary Material 17.

Supplementary Material 18.

Supplementary Material 19.

Supplementary Material 20.

Supplementary Material 21.

Supplementary Material 22.

Supplementary Material 23.

## Data Availability

The genetic and metabolic data that support the findings of this study are available from UK Biobank, but restrictions apply to the availability of these data. However, data are available following registration with and permission of UK Biobank. The GWAS data are publicly available and are either included in their corresponding published articles or the NHGRI-EBI GWAS Catalog. All scripts used in our analyses are publicly available in our GitHub repository: [https://github.gatech.edu/jkonarkowski3/BCAA](https:/github.gatech.edu/jkonarkowski3/BCAA).

## Abbreviations

BCAAs: branched-chain amino acids (Leucine, Isoleucine, and Valine)
BCAT: branched-chain amino acid aminotransferase
BMI: body-mass index
CKD: chronic kidney disease
EAF: estimated allele frequency
eGFR: estimated glomerular filtration rate
GWAS: genome-wide association study
ICD: international classification of diseases
IVW: inverse variance weighted
LD: linkage disequilibrium
MR: Mendelian randomization
MSUD: maple syrup urine disease
PGS: polygenic score
PheWAS: phenome-wide association study
PRS: polygenic risk score (used specifically to refer to risk for disease)
PRS-CS: polygenic risk score-continuous shrinkage
SA: sleep apnea
SBP: systolic blood pressure
SNP: single nucleotide polymorphism
T2D: type II diabetes
UKB: UK Biobank
