# Supplementary material for: Assessment of genetic and metabolite associations of branched chain amino acids with metabolic disease in the UK Biobank using Mendelian randomization
Source: BMC Med Genomics. 2025 Oct 16;18:163. doi: 10.1186/s12920-025-02232-2 (PMC12532399; doi:10.1186/s12920-025-02232-2)
Supplement: Supplementary file 1 — Supplementary Material 1. [file 12920_2025_2232_MOESM1_ESM.docx]

**Supplementary Figures**

**Supplementary Figure 1** Disease associations within the significant threshold for leucine (A), isoleucine (B), and valine (C), respectively, plotted against log(p-value).

**Supplementary Figure 2** MR Scatter Plots for BCAAs and BMI, with each line representing a different MR method and dots representing individual instruments. Plots with BCAAs as exposure are on the left, and plots with disease as exposure are on the right.

**Supplementary Figure 3** MR Scatter Plots for BCAAs and eGFR, with each line representing a different MR method and dots representing individual instruments. Plots with BCAAs as exposure are on the left, and plots with disease as exposure are on the right.

**Supplementary Figure 4** MR Scatter Plots for BCAAs and Sleep Apnea, with each line representing a different MR method and dots representing individual instruments. Plots with BCAAs as exposure are on the left, and plots with disease as exposure are on the right.

**Supplementary Figure 5** MR Scatter Plots for BCAAs and Systolic Blood Pressure, with each line representing a different MR method and dots representing individual instruments. Plots with BCAAs as exposure are on the left, and plots with disease as exposure are on the right.

**Supplementary Figure 6** MR Scatter Plots for BCAAs and Type II Diabetes, with each line representing a different MR method and dots representing individual instruments.

**Supplementary Tables**

**Supplementary Table 1**

1. ICD9 – ICD9 code
2. ICD9 String – Standard name for disease corresponding to ICD9 code
3. PheCode – code grouping ICD9 codes into broader phenotypes
4. Phenotype – broader phenotype name associated with PheCode

**Supplementary Table 2**

1. phecode_id - code grouping ICD9 codes into broader phenotypes
2. disease_name – name of phenotype corresponding to the specific PheCode
3. total_n_samples – number of individuals with data for given PheCode
4. n_case_samples – number of individuals who have the associated phenotype
5. n_contol_samples – number of individuals who do not have the phenotype
6. estimate – change in the log-odds of phenotype for each unit increase of BCAA
7. se – standard error for estimate
8. z – normalized value of estimate (estimate/se)
9. lo_ci – lower bound of 95% confidence interval for odds ratio
10. hi_ci – upper bound of 95% confidence interval for odds ratio
11. or – odds ratio for given phenotype
12. pval – p-value showing statistical significance of the association

**Supplementary Table 3**

1. chr – chromosome where SNP is located
2. rsid – SNP identifier
3. bp – base pair position of SNP on chromosome
4. effect_allele – one of the possible alleles at the specific SNP of which the effect is being measured
5. other_allele – the alternate possible allele at the specific SNP
6. weight – SNP weight for PRS

**Supplementary Table 4**

1. SNP – rsID SNP identifier
2. effect_allele – one of the possible alleles at the specific SNP of which the effect is being measured
3. other_allele – the alternate possible allele at the specific SNP
4. beta – log-odds value showing size and direction of effect of allele on outcome
5. se – standard error of beta
6. pval – p-value showing statistical significance of the GWAS association. SNPs were included as instruments in each MR if the value in the relevant column for the specific MR analysis was less than the genome-wide significance threshold of p<5E-08
7. eaf – effect allele frequency in study sample
8. Chromosome – chromosome where SNP is located
9. Position – position of SNP on chromosome

**Supplementary Table 5**

1. id.exposure – unique identifier of exposure variable generated by TwoSampleMR
2. id.outcome – unique identifier of outcome variable generated by TwoSampleMR
3. outcome – name of outcome variable
4. exposure – name of exposure variable
5. method – specific MR method used for analysis
6. nsnp – number of SNPs used by the MR method
7. b –log-odds effect size of exposure on outcome
8. se – standard error of beta
9. pval – p-value showing statistical significance of the association

**Supplementary Table 6**

1. exposure – name of exposure variable
2. outcome – name of outcome variable
3. id.exposure– unique identifier of exposure variable generated by TwoSampleMR
4. id.outcome – unique identifier of outcome variable generated by TwoSampleMR
5. SNP – rsID SNP identifier
6. b – log-odds effect size of exposure on outcome
7. se – standard error of beta
8. p – p-value showing statistical significance of the association
9. Chromosome – chromosome where SNP is located
10. Position – position of SNP on chromosome

**Supplementary Table 7**

1. exposure –name of exposure variable
2. outcome – name of outcome variable
3. id.exposure – unique identifier for the exposure variable generated by TwoSampleMR
4. id.outcome – unique identifier for the outcome variable generated by TwoSampleMR
5. Q – Cochran's Q, measuring the total heterogeneity among SNPs
6. Q_df – degrees of freedom, calculated as the number of SNPs minus one
7. Q_pval – p-value for the Q test, low indicates high heterogeneity

**Supplementary Table 8**

1. exposure – name of exposure variable
2. outcome – name of outcome variable
3. analysis – name of analysis type; raw includes all SNPs before outlier correction
4. b – log-odds effect size of exposure on outcome
5. se – standard error of beta
6. tstat -- test statistic for the null hypothesis that beta is 0
7. pval – p-value showing statistical significance of the association
8. global_RSSobs – observed residual sum of squares from global test
9. global_pval – p-value for global test
10. distortion_coeff – difference in causal estimate before/after outlier removal
11. distortion_pval – p-value for the distortion test
12. n_outliers – number of outlier SNPs removed in corrected model

In addition to these supplementary tables and figures, we have included the STROBE-MR checklist in our supplementary documents.
