# Supplementary material for: Assessment of genetic and metabolite associations of branched chain amino acids with metabolic disease in the UK Biobank using Mendelian randomization
Source: BMC Med Genomics. 2025 Oct 16;18:163. doi: 10.1186/s12920-025-02232-2 (PMC12532399; doi:10.1186/s12920-025-02232-2)
Supplement: Supplementary file 2 — Supplementary Material 2. [file 12920_2025_2232_MOESM2_ESM.docx]

**STROBE-MR checklist of recommended items to address in reports of Mendelian randomization studies**^1^ ^2^

| **Item No.** | **Section** | **Checklist item** | **Page No.** | **Relevant text from manuscript** |
| --- | --- | --- | --- | --- |
| 1 | **TITLE and ABSTRACT** | Indicate Mendelian randomization (MR) as the study’s design in the title and/or the abstract if that is a main purpose of the study | 1 | “Assessment of Genetic and Metabolite Associations of Branched Chain Amino Acids with Metabolic Disease in the UK Biobank using Mendelian Randomization” |
|  | **INTRODUCTION** |  |  |  |
| 2 | **Background** | Explain the scientific background and rationale for the reported study. What is the exposure? Is a potential causal relationship between exposure and outcome plausible? Justify why MR is a helpful method to address the study question | 3, 5 | “Due to their involvement in a multitude of important metabolic pathways and ability to be quantified in blood testing, several correlational associations between BCAA levels and disease have been identified”  “Causal effects of BCAA levels have been inferred with variable strength of evidence in cardiovascular disease (24,25,22,26,27), hypertension (28,29), Alzheimer’s disease (30,31), specific cancers (32, 33, 34), type II diabetes (35,36), and obesity (26).” |
| 3 | **Objectives** | State specific objectives clearly, including pre-specified causal hypotheses (if any). State that MR is a method that, under specific assumptions, intends to estimate causal effects | 4 | “Many recent studies have employed Mendelian Randomization (MR), a statistical method that treats genetic variants as natural experiments to investigate whether a specific factor causally affects disease status (22). This approach is based on the principle that genetic variants are randomly assigned at conception and are therefore not influenced by lifestyle or environmental factors, reducing confounding and reverse causation, and providing a plausible inference of causality (23).” |
|  | **METHODS** |  |  |  |
| 4 | **Study design and data sources** | Present key elements of the study design early in the article. Consider including a table listing sources of data for all phases of the study. For each data source contributing to the analysis, describe the following: |  |  |
|  | a) | Setting: Describe the study design and the underlying population, if possible. Describe the setting, locations, and relevant dates, including periods of recruitment, exposure, follow-up, and data collection, when available. | 6 | “Our analysis utilized the UK Biobank (UKB) cohort (39) consisting of 502,364 individuals in the United Kingdom” |
|  | b) | Participants: Give the eligibility criteria, and the sources and methods of selection of participants. Report the sample size, and whether any power or sample size calculations were carried out prior to the main analysis | 6 | “Following data filtering and quality control, data associated with 152,199 unique individuals was used in the final study, namely 30.3% of the total sample. The exclusion criteria were violation of the following: kinship found (ID: 22021; ~150,000 excluded), White British only (ID: 22006; n=409,551), self-reported sex matches genetic sex (ID: 22001, 31), no sex chromosome aneuploidy (ID: 22019), no information present for age (ID: 21022), no information present for sex (ID:31), and no information present for one or more of the covariates (ID:22009). A further approximately 130,000 individuals were excluded because there was no available metabolite data for leucine (ID: 23466), isoleucine (ID:23465), or valine (ID: 23467). The primary phenotypic data utilized in our analyses consisted of the aforementioned BCAA levels as well as disease case/control status.” |
|  | c) | Describe measurement, quality control and selection of genetic variants | 6-7 | “The genotypic component of our study included the calculation of polygenic scores (PGS) for leucine, isoleucine, and valine using data sourced from a 2024 consortium study covering approximately 12 million SNPs in over 135,000 individuals who did not overlap with the UK Biobank, instead relying on 33 cohorts primarily from continental Europe (120,241 individuals/ 88.4% of total), China (4,435 individuals/ 3.3%), and South Asia (11,340 individuals/ 8.3%) (21). “ |
|  | d) | For each exposure, outcome, and other relevant variables, describe methods of assessment and diagnostic criteria for diseases | 10-11 | “GWAS summary statistics for diseases were sourced from several different publications. Type II Diabetes GWAS data was sourced from the European-ancestry dataset from Mahjan and the DIAMANTE consortium (43), with a sample of 933,970 individuals from 32 different cohorts, of which 80,154 were cases and 853,816 were controls based on ICD codes for T2D or T2D complications in participant health records or purchases of T2D treatment drugs. Sleep apnea (SA) GWAS data was sourced from Campos et al (44) and consisted of 362,638 European-ancestry individuals from five different datasets: UK Biobank, the Canadian Longitudinal Study of Aging, the Australian Genetics of Depression Study, Partners Healthcare Biobank, and FinnGen. There were 25,008 cases and 337,630 controls, and disease status was either self-reported or sourced from ICD codes in electronic health records.    In some cases, studies of continuous traits closely linked to the disease were used instead of the disease itself due to greater statistical power resulting from a larger sample size. The systolic blood pressure (SBP) GWAS from Keaton et al (45) was utilized for hypertension due to its sample size of 1,028,980, the largest of any such study to date. The participants were all of European ancestry and were sourced from UK Biobank, the International Consortium for Blood Pressure, Million Veteran Program and BioVU. The Stanzick et al GWAS of estimated glomerular filtration rate (eGFR) (46) was used for CKD, specifically the EUR dataset consisting of 1,004,040 European-ancestry participants sourced from the CKDGen Consortium and UK Biobank. The body mass index (BMI) GWAS from Pulit et al (47) was utilized for obesity, consisting of 694,649 European-ancestry participants from the GIANT Consortium and UK Biobank.” |
|  | e) | Provide details of ethics committee approval and participant informed consent, if relevant | 33 | " Approval to access UK Biobank data was provided under project number 17984 to GG with the permission of the Georgia Institute of Technology IRB. Participants consent to approved use of metabolite and genetic data.” |
| 5 | **Assumptions** | Explicitly state the three core IV assumptions for the main analysis (relevance, independence and exclusion restriction) as well assumptions for any additional or sensitivity analysis | 11 | “Our MR analyses rely on three core instrumental variable assumptions. The first assumption is relevance, which assumes that genetic variants are robustly associated with the exposure. The second is independence, which assumes that there are no confounders of the instrument-outcome relationship. The final assumption is exclusion restriction, that instruments only influence the outcome through the exposure.” |
| 6 | **Statistical methods: main analysis** | Describe statistical methods and statistics used |  |  |
|  | a) | Describe how quantitative variables were handled in the analyses (i.e., scale, units, model) | 11 | “Prior to conducting any analyses, each dataset was cleaned and reformatted to a common column standard (SNP, effect_allele, other_allele, beta, se, pval, eaf) to ensure compatibility with downstream data processing scripts” |
|  | b) | Describe how genetic variants were handled in the analyses and, if applicable, how their weights were selected | 11 | “For the exposure data, we selected the genetic variants associated with the trait at genome-wide significance (p < 5x10⁻⁸) and applied linkage disequilibrium (LD) pruning (r² < 0.001 within a 10Mb window) using 1000Genomes EUR LD blocks (48) and PLINK 1.9 beta 7.7 (49) to ensure independence of instruments for MR. For the outcome data, we retained all SNPs to maximize overlap during harmonization. We performed all MR in R 4.5.0 using a custom script utilizing the TwoSampleMR package v0.6.15 (50).” |
|  | c) | Describe the MR estimator (e.g. two-stage least squares, Wald ratio) and related statistics. Detail the included covariates and, in case of two-sample MR, whether the same covariate set was used for adjustment in the two samples | 11-12,  12 | “MR was conducted using inverse variance weighted (IVW) as the primary analysis for association discovery. Simple mode, weighted mode, MR-Egger and weighted median were conducted as well to assess the robustness of the analysis under weaker assumptions.” “As we utilized GWAS data that had been adjusted for covariates during primary association testing, no further adjustment was conducted along our MR pipeline.” |
|  | d) | Explain how missing data were addressed | 11 | “First, exposure and outcome datasets were harmonized to align effect alleles and remove missing data as well as ambiguous or palindromic SNPs with an EAF between 0.42 and 0.58.” |
|  | e) | If applicable, indicate how multiple testing was addressed | 13  21 | " Initial PheWAS analysis identified disease associations with p-values ranging from the Bonferroni-adjusted significance threshold of 1×10-5 to around 1 ×10-187for the associations with the strongest evidence against the null hypothesis.”  “In the forward MR direction, where BCAAs were the exposure and diseases were the outcome, most associations showed either no or weak evidence of causality (Figure 4), with the exception of isoleucine and SBP (β = 1.62; SE: 0.44; p = 2.14×10⁻⁴) and leucine and SBP (β = 1.79; SE: 0.55; p = 0.001), both of which showed positive associations. A much weaker effect was observed between valine and SBP (β = 0.64; SE: 0.41; p = 0.114).” |
| 7 | **Assessment of assumptions** | Describe any methods or prior knowledge used to assess the assumptions or justify their validity | 12 | “Simple mode, weighted mode, MR-Egger and weighted median were conducted as well to assess the robustness of the analysis under weaker assumptions.” |
| 8 | **Sensitivity analyses and additional analyses** | Describe any sensitivity analyses or additional analyses performed (e.g. comparison of effect estimates from different approaches, independent replication, bias analytic techniques, validation of instruments, simulations) | 22 | “We also performed Cochran’s Q tests to assess heterogeneity and MR-PRESSO to identify and correct outlier instruments in our initial analysis, and these additional analyses are reported in Supplementary Tables 8 and 9 respectively.” |
| 9 | **Software and pre-registration** |  |  |  |
|  | a) | Name statistical software and package(s), including version and settings used | 11 | “We performed all MR in R 4.5.0 using a custom script utilizing the TwoSampleMR package v0.6.15 (50).” |
|  | b) | State whether the study protocol and details were pre-registered (as well as when and where) |  | N/A |
|  | **RESULTS** |  |  |  |
| 10 | **Descriptive data** |  |  |  |
|  | a) | Report the numbers of individuals at each stage of included studies and reasons for exclusion. Consider use of a flow diagram | 6 | “The exclusion criteria were violation of the following: kinship found (ID: 22021; ~150,000 excluded), White British only (ID: 22006; n=409,551), self-reported sex matches genetic sex (ID: 22001, 31), no sex chromosome aneuploidy (ID: 22019), no information present for age (ID: 21022), no information present for sex (ID:31), and no information present for one or more of the covariates (ID:22009)” |
|  | b) | Report summary statistics for phenotypic exposure(s), outcome(s), and other relevant variables (e.g. means, SDs, proportions) | 13 | “The effect sizes (β) and corresponding standard errors (SE) for these associations ranged from β = -0.38 to 0.58 (SE: 0.0068-0.0894) for leucine, β = -0.38 to 0.58 (SE: 0.0068-0.0894) for isoleucine, and β = -0.27 to 0.53 (SE: 0.0069-0.0892) for valine. There was a high degree of similarity between leucine and isoleucine in disease associations, with 19 associations shared between the two and not shared with valine. For all three BCAAs, the vast majority of associations indicate a positive relationship where higher circulating metabolite levels correspond to higher prevalence of disease (40/62 for Leu, 40/59 for Ile, and 45/51 for Val; Figure 1B, C, D). A full listing and visualization of metabolite-disease associations within our significance threshold is provided in Supplementary Table 3 and Supplementary Figure 1, while the full PheWAS with all diseases is in Supplementary Table 2.” |
|  | c) | If the data sources include meta-analyses of previous studies, provide the assessments of heterogeneity across these studies |  | N/A |
|  | d) | For two-sample MR:  i.  Provide justification of the similarity of the genetic variant-exposure associations between the exposure and outcome samples  ii.  Provide information on the number of individuals who overlap between the exposure and outcome studies | 28  6 | “A major limitation of our study is that our conclusions are not necessarily transferable to other population groups due to a lack of inclusion of non-European ancestry individuals in our analyses”  “The genotypic component of our study included the calculation of polygenic scores (PGS) for leucine, isoleucine, and valine using data sourced from a 2024 consortium study covering approximately 12 million SNPs in over 135,000 individuals who did not overlap with the UK Biobank,” |
| 11 | **Main results** |  |  |  |
|  | a) | Report the associations between genetic variant and exposure, and between genetic variant and outcome, preferably on an interpretable scale |  | See Supplementary Table 7 |
|  | b) | Report MR estimates of the relationship between exposure and outcome, and the measures of uncertainty from the MR analysis, on an interpretable scale, such as odds ratio or relative risk per SD difference | 20-21 | “In the forward MR direction, where BCAAs were the exposure and diseases were the outcome, most associations showed either no or weak evidence of causality (Figure 4), with the exception of isoleucine and SBP (β = 1.62; SE: 0.44; p = 2.14×10⁻⁴) and leucine and SBP (β = 1.79; SE: 0.55; p = 0.001), both of which showed positive associations. A much weaker effect was observed between valine and SBP (β = 0.64; SE: 0.41; p = 0.114).    In the reverse direction, with disease as the exposure and BCAAs as the outcome, stronger and more consistent effects were observed (Figure 5). There is strong evidence for an effect of type 2 diabetes (T2D) liability on all three BCAAs (see next section), including valine (β = 0.07; SE: 0.01; p = 8.59×10⁻¹⁴), isoleucine (β = 0.07; SE: 0.01; p = 7.14×10⁻⁸), and leucine (β = 0.08; SE: 0.01; p = 1.94×10⁻⁹). We also found suggestive evidence for associations of sleep apnea liability with valine levels (β = 0.14; SE: 0.06; p = 0.016). In these analyses, the beta coefficient (β) represents the standard deviation (SD) change in BCAA levels per one-unit increase in the log-odds of the genetically predicted disease, interpreted as a life-course effect of disease liability.” |
|  | c) | If relevant, consider translating estimates of relative risk into absolute risk for a meaningful time period | 11 | " In the case that an individual had multiple reported values of a metabolite due to data collection at two separate times, the median of the two values was utilized in the z-score calculation.” |
|  | d) | Consider plots to visualize results (e.g. forest plot, scatterplot of associations between genetic variants and outcome versus between genetic variants and exposure) | 19-20,24 | Figure 4, Figure 5 (Forest Plot)  Figure 7 (Scatterplot) |
| 12 | **Assessment of assumptions** |  |  |  |
|  | a) | Report the assessment of the validity of the assumptions | 22 | “Additional methods such as simple mode and weighted mode as well as sensitivity analyses (MR-Egger, weighted median) are reported in Supplementary Table 6 and Supplementary Figures 2-6 with results on the individual SNP level in Supplementary Table 7 and are generally consistent with the main findings. MR-Egger intercept tests did not indicate significant directional pleiotropy for most metabolite-disease pairs (all p > 0.05), apart from Ile to eGFR (p = 2.9×10⁻⁴), Leu to eGFR (p = 0.0096), and T2D to Val (p = 0.017) relationships. We also performed Cochran’s Q tests to assess heterogeneity and MR-PRESSO to identify and correct outlier instruments in our initial analysis, and these additional analyses are reported in Supplementary Tables 8 and 9 respectively.” |
|  | b) | Report any additional statistics (e.g., assessments of heterogeneity across genetic variants, such as *I^2^*, Q statistic or E-value) | 22 | “We found the Ile to SBP pairing to have high heterogeneity (Q_IVW = 428.65; Q_Egger = 420.57), and MR-PRESSO revealed a decrease in the causal estimate after removing outliers (β_raw = 1.72; β_corrected = 1.29). Similarly, Leu to SBP (Q_IVW = 377.32; Q_Egger = 359.01), and Ile to T2D demonstrated very high heterogeneity (Q_IVW = 945.04; Q_Egger = 866.33). In the reverse direction, pairings such as Ile to eGFR (Q_IVW = 491.15; MR-PRESSO β_raw = 0.011; β_corrected = 0.0054) and Leu to BMI (Q_IVW = 323.42; MR-PRESSO β_raw = –0.012; β_corrected = 0.049) exhibited both heterogeneity and major changes in effect size following outlier removal, further supporting the presence of pleiotropic distortion” |
| 13 | **Sensitivity analyses and additional analyses** |  |  |  |
|  | a) | Report any sensitivity analyses to assess the robustness of the main results to violations of the assumptions | 22 | “We found the Ile to SBP pairing to have high heterogeneity (Q_IVW = 428.65; Q_Egger = 420.57), and MR-PRESSO revealed a decrease in the causal estimate after removing outliers (β_raw = 1.72; β_corrected = 1.29). Similarly, Leu to SBP (Q_IVW = 377.32; Q_Egger = 359.01), and Ile to T2D demonstrated very high heterogeneity (Q_IVW = 945.04; Q_Egger = 866.33). In the reverse direction, pairings such as Ile to eGFR (Q_IVW = 491.15; MR-PRESSO β_raw = 0.011; β_corrected = 0.0054) and Leu to BMI (Q_IVW = 323.42; MR-PRESSO β_raw = –0.012; β_corrected = 0.049) exhibited both heterogeneity and major changes in effect size following outlier removal, further supporting the presence of pleiotropic distortion” |
|  | b) | Report results from other sensitivity analyses or additional analyses | 22 | “Additional methods such as simple mode and weighted mode as well as sensitivity analyses (MR-Egger, weighted median) are reported in Supplementary Table 6 and Supplementary Figures 2-6 with results on the individual SNP level in Supplementary Table 7 and are generally consistent with the main findings.” |
|  | c) | Report any assessment of direction of causal relationship (e.g., bidirectional MR) | 26-27 | “Broadly, our MR results present a more complex picture, namely that while BCAAs like leucine and isoleucine have some causal effect on blood pressure and hypertension, other diseases such as type II diabetes show a reverse causal effect where disease modulates BCAA levels while BCAA levels have little effect on causing the disease. Our analyses also show limited evidence for positive causality of sleep apnea on valine levels or isoleucine on hypertension.” |
|  | d) | When relevant, report and compare with estimates from non-MR analyses | 26 | “While our observational and PGS analyses suggested strong associations between BCAAs and metabolic diseases and confirmed that genetics plays a role in these relationships, MR provides further clarification by attempting to disentangle correlation from causation.” |
|  | e) | Consider additional plots to visualize results (e.g., leave-one-out analyses) |  | See Supplementary Figures 2-6 |
|  | **DISCUSSION** |  |  |  |
| 14 | **Key results** | Summarize key results with reference to study objectives | 26-27 | “Broadly, our MR results present a more complex picture, namely that while BCAAs like leucine and isoleucine have some causal effect on blood pressure and hypertension, other diseases such as type II diabetes show a reverse causal effect where disease modulates BCAA levels while BCAA levels have little effect on causing the disease. Our analyses also show limited evidence for positive causality of sleep apnea on valine levels or isoleucine on hypertension.” |
| 15 | **Limitations** | Discuss limitations of the study, taking into account the validity of the IV assumptions, other sources of potential bias, and imprecision. Discuss both direction and magnitude of any potential bias and any efforts to address them | 27 | “However, the robustness of these relationships is somewhat challenged by high heterogeneity, implying horizontal pleiotropy, though MR-Egger shows that most of the findings remain robust.” |
| 16 | **Interpretation** |  |  |  |
|  | a) | Meaning: Give a cautious overall interpretation of results in the context of their limitations and in comparison with other studies | 27 | “Overall, our MR clearly demonstrates how positive associations between a metabolite level or metabolite PGS and disease prevalence may not imply causation, rather simply reflecting how genetic associations with the metabolite maybe confounded by the influence of disease in actually increasing the BCAA.” |
|  | b) | Mechanism: Discuss underlying biological mechanisms that could drive a potential causal relationship between the investigated exposure and the outcome, and whether the gene-environment equivalence assumption is reasonable. Use causal language carefully, clarifying that IV estimates may provide causal effects only under certain assumptions | 28 | “The role of BCAAs in these relationships is not fully understood, though a potential explanation could involve the ability of BCAAs to induce insulin release, which leads to an increase in overall blood pressure (28). The peripheral circulatory manifestations are likely linked to this causal mechanism as well, and this explanation would be consistent with our MR results. It is possible that similar biological mechanisms may contribute to the renal manifestations. Alternatively, elevated BCAAs might contribute more directly to renal issues by promoting inflammation, oxidative stress, or mitochondrial dysfunction, processes commonly linked to diabetic nephropathy. The latter explanation is supported by previous animal testing, which found increased CKD progression in rats in an amino-acid rich diet (54), though these findings are not fully generalizable to CKD in humans and our MR does not support this explanation.” |
|  | c) | Clinical relevance: Discuss whether the results have clinical or public policy relevance, and to what extent they inform effect sizes of possible interventions | 28 | “Our work not only identifies chronic kidney disease (CKD), obesity, hypertension, and sleep apnea as diseases of interest related to BCAAs but also highlights the potential for PheWAS to uncover novel and specific associations, such as the link between BCAAs and type II diabetes with renal manifestations. By pinpointing this relationship, we underscore the ability of large-scale PheWAS to provide nuanced insights into how metabolic pathways contribute to disease subtypes, which may otherwise remain obscured in broader analyses. These findings illustrate the power of PheWAS techniques as a powerful tool for generating hypotheses for future functional studies, guiding biomarker discovery, and identifying potential therapeutic targets for complex disease.” |
| 17 | **Generalizability** | Discuss the generalizability of the study results (a) to other populations, (b) across other exposure periods/timings, and (c) across other levels of exposure | 28 | “A major limitation of our study is that our conclusions are not necessarily transferable to other population groups due to a lack of inclusion of non-European ancestry individuals in our analyses, owing to low sample size and efforts to avoid potential population stratification.” |
|  | **OTHER INFORMATION** |  |  |  |
| 18 | **Funding** | Describe sources of funding and the role of funders in the present study and, if applicable, sources of funding for the databases and original study or studies on which the present study is based | 32 | “No funding was used to support this project.” |
| 19 | **Data and data sharing** | Provide the data used to perform all analyses or report where and how the data can be accessed, and reference these sources in the article. Provide the statistical code needed to reproduce the results in the article, or report whether the code is publicly accessible and if so, where | 31 | “The genetic and metabolic data that support the findings of this study are available from UK Biobank, but restrictions apply to the availability of these data. However, data are available following registration with and permission of UK Biobank. The GWAS data are publicly available and are either included in their corresponding published articles or the NHGRI-EBI GWAS Catalog. All scripts used in our analyses are publicly available in our GitHub repository: https://github.gatech.edu/jkonarkowski3/BCAA” |
| 20 | **Conflicts of Interest** | All authors should declare all potential conflicts of interest | 32 | “The authors declare that they do not have any competing interests or conflicts.” |

This checklist is copyrighted by the Equator Network under the Creative Commons Attribution 3.0 Unported (CC BY 3.0) license.

1. Skrivankova VW, Richmond RC, Woolf BAR, Yarmolinsky J, Davies NM, Swanson SA, et al. Strengthening the Reporting of Observational Studies in Epidemiology using Mendelian Randomization (STROBE-MR) Statement. JAMA. 2021;under review.

2. Skrivankova VW, Richmond RC, Woolf BAR, Davies NM, Swanson SA, VanderWeele TJ, et al. Strengthening the Reporting of Observational Studies in Epidemiology using Mendelian Randomisation (STROBE-MR): Explanation and Elaboration. BMJ. 2021;375:n2233.
