# Supplementary material for: Assessment of genetic and metabolite associations of branched chain amino acids with metabolic disease in the UK Biobank using Mendelian randomization
Source: BMC Med Genomics. 2025 Oct 16;18:163. doi: 10.1186/s12920-025-02232-2 (PMC12532399; doi:10.1186/s12920-025-02232-2)
Supplement: Supplementary file 3 — Supplementary Material 3. [file 12920_2025_2232_MOESM3_ESM.pdf]

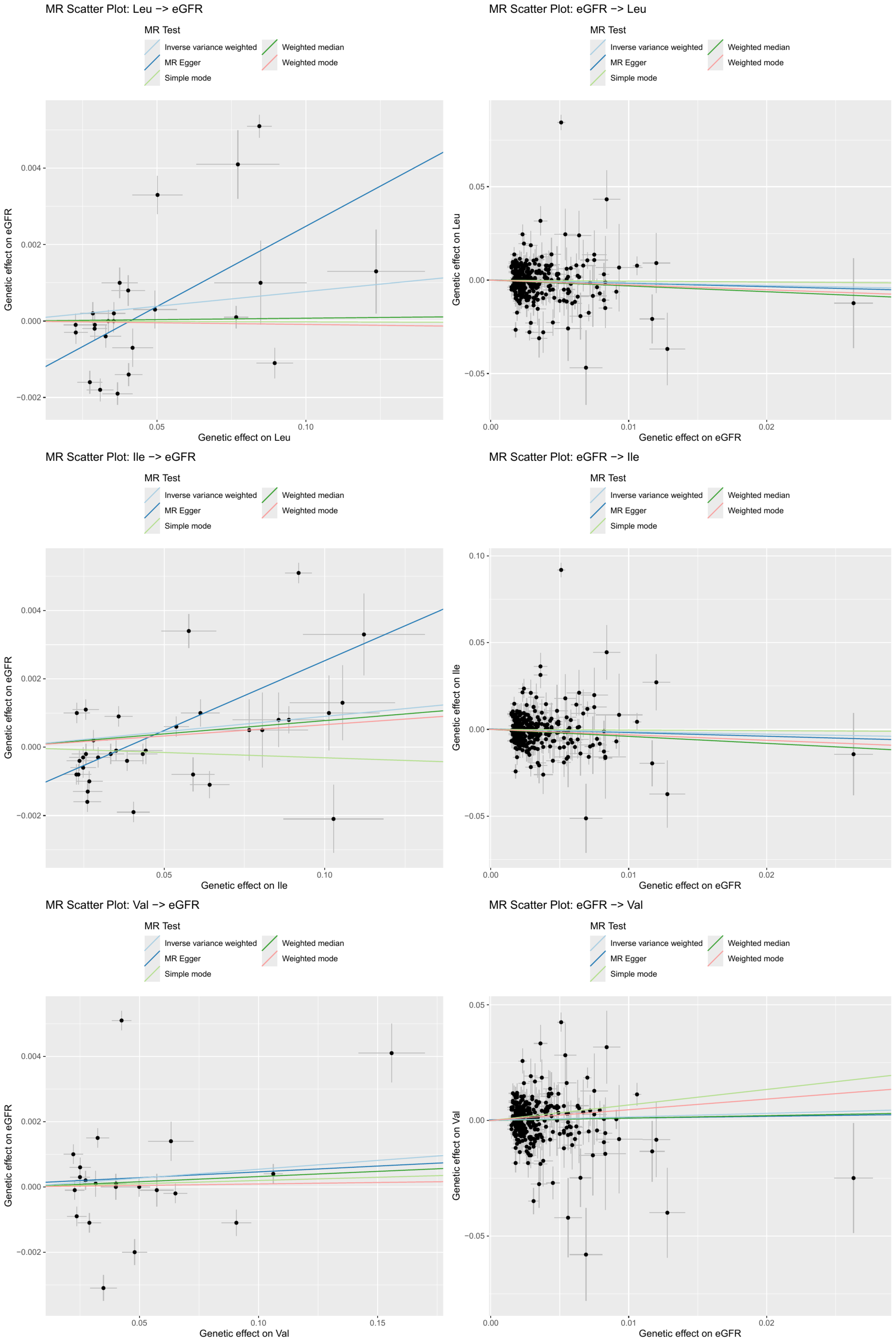
MR Scatter Plot: Leu −> eGFR
MR Test
Inverse variance weighted
MR Egger
Simple mode
Weighted median
Weighted mode
Genetic effect on eGFR
Genetic effect on Leu
0.004
0.002
0.000
−0.002
0.05
0.10
MR Scatter Plot: eGFR −> Leu
MR Test
Inverse variance weighted
MR Egger
Simple mode
Weighted median
Weighted mode
Genetic effect on Leu
Genetic effect on eGFR
0.05
0.00
−0.05
0.00
0.01
0.02
MR Scatter Plot: Ile −> eGFR
MR Test
Inverse variance weighted
MR Egger
Simple mode
Weighted median
Weighted mode
Genetic effect on eGFR
Genetic effect on Ile
0.004
0.002
0.000
−0.002
0.05
0.10
MR Scatter Plot: eGFR −> Ile
MR Test
Inverse variance weighted
MR Egger
Simple mode
Weighted median
Weighted mode
Genetic effect on Ile
Genetic effect on eGFR
0.10
0.05
0.00
−0.05
0.00
0.01
0.02
MR Scatter Plot: Val −> eGFR
MR Test
Inverse variance weighted
MR Egger
Simple mode
Weighted median
Weighted mode
Genetic effect on eGFR
Genetic effect on Val
0.004
0.002
0.000
−0.002
0.05
0.10
0.15
MR Scatter Plot: eGFR −> Val
MR Test
Inverse variance weighted
MR Egger
Simple mode
Weighted median
Weighted mode
Genetic effect on Val
Genetic effect on eGFR
0.05
0.00
−0.05
0.00
0.01
0.02
