# Supplementary figures and images for: Assessment of genetic and metabolite associations of branched chain amino acids with metabolic disease in the UK Biobank using Mendelian randomization
Source: BMC Med Genomics. 2025 Oct 16;18:163. doi: 10.1186/s12920-025-02232-2 (PMC12532399; doi:10.1186/s12920-025-02232-2)

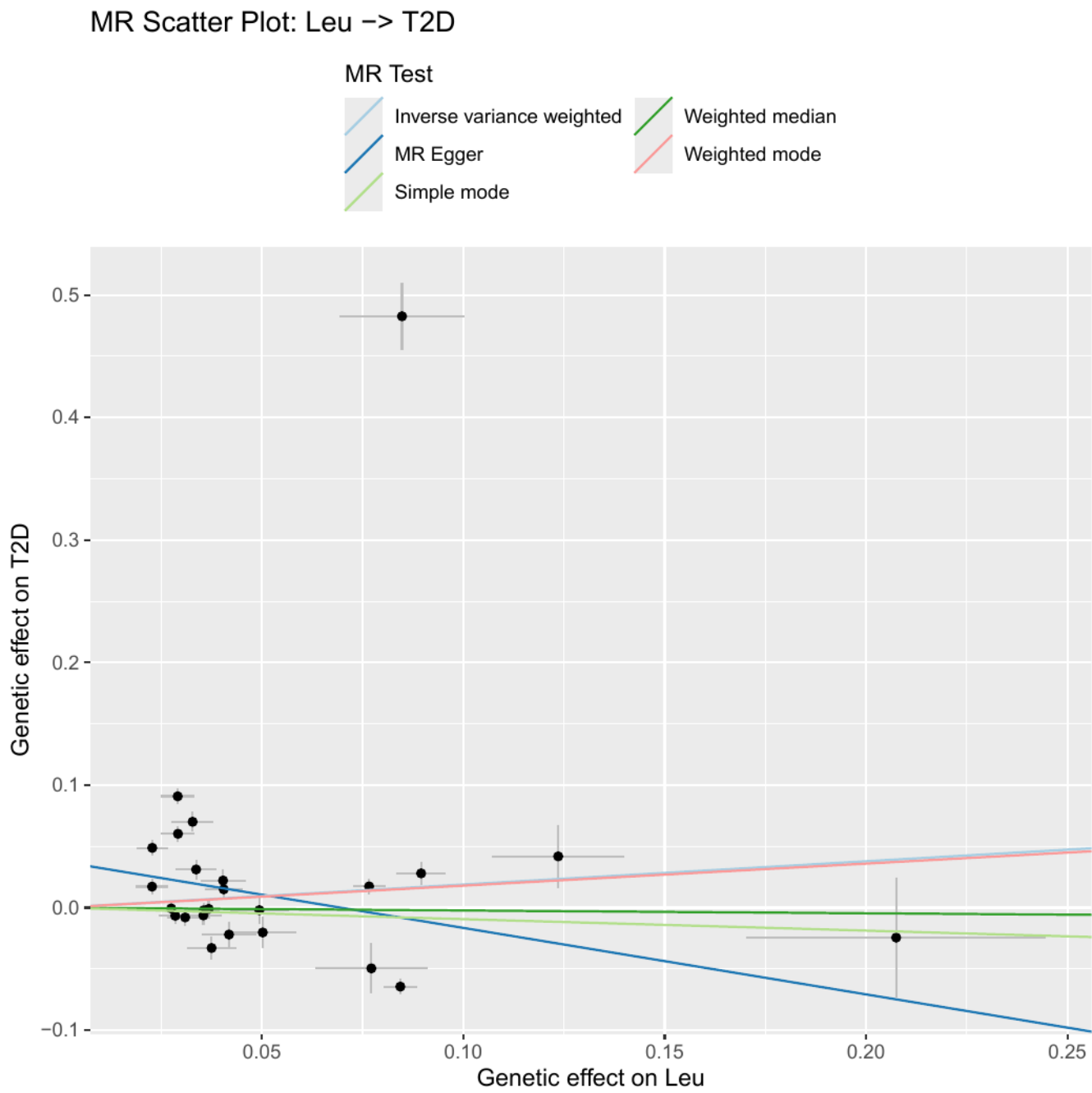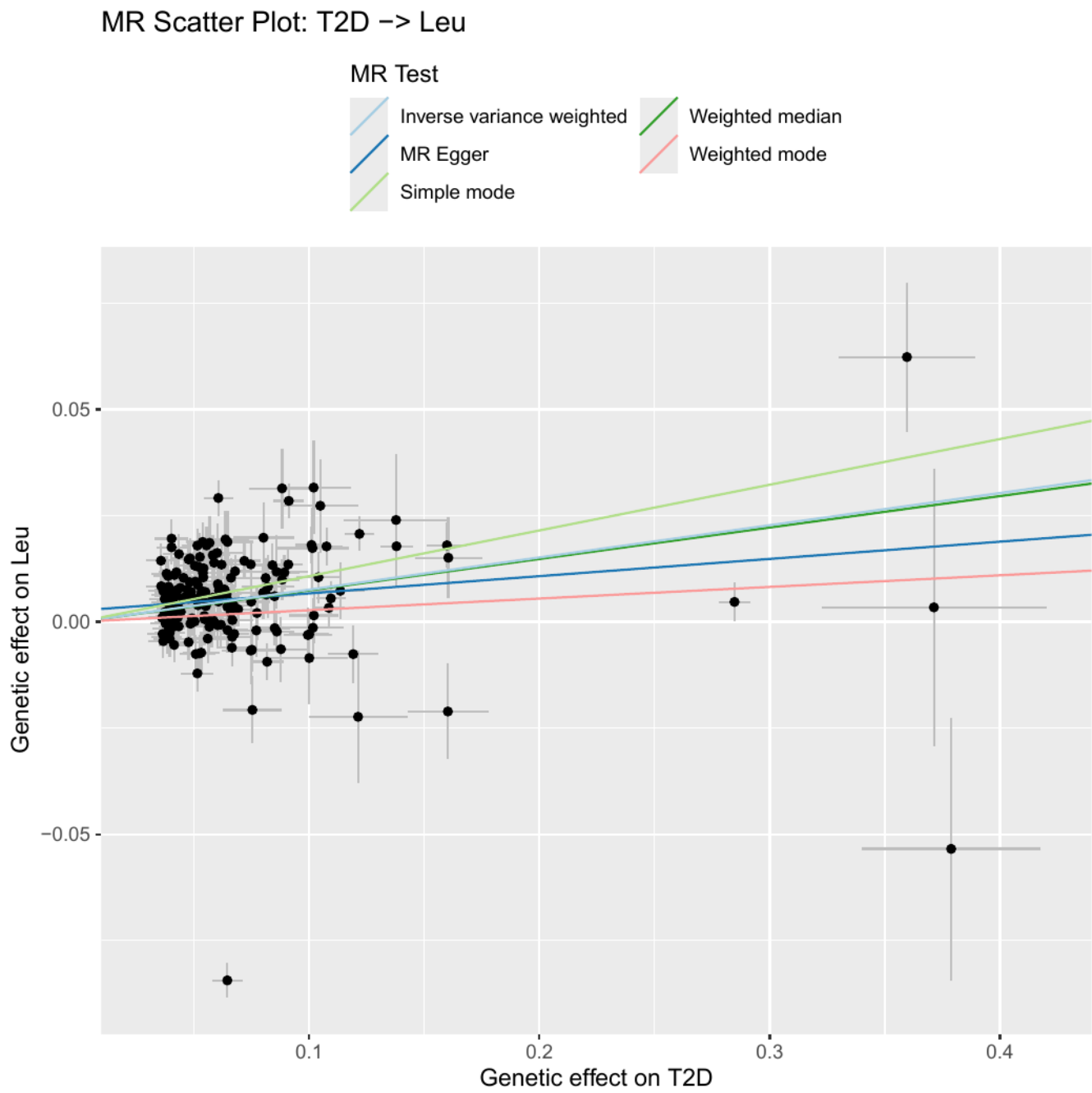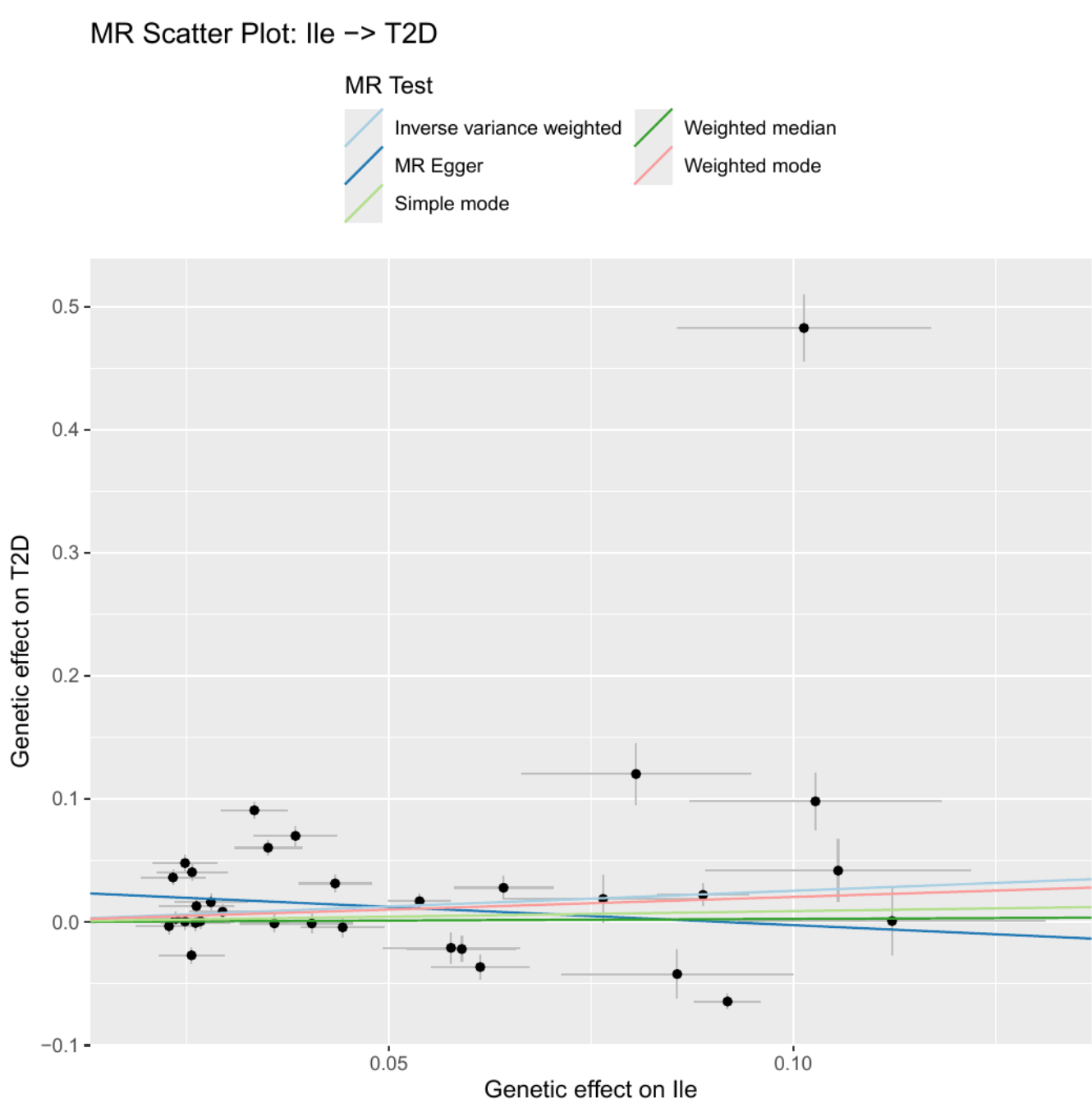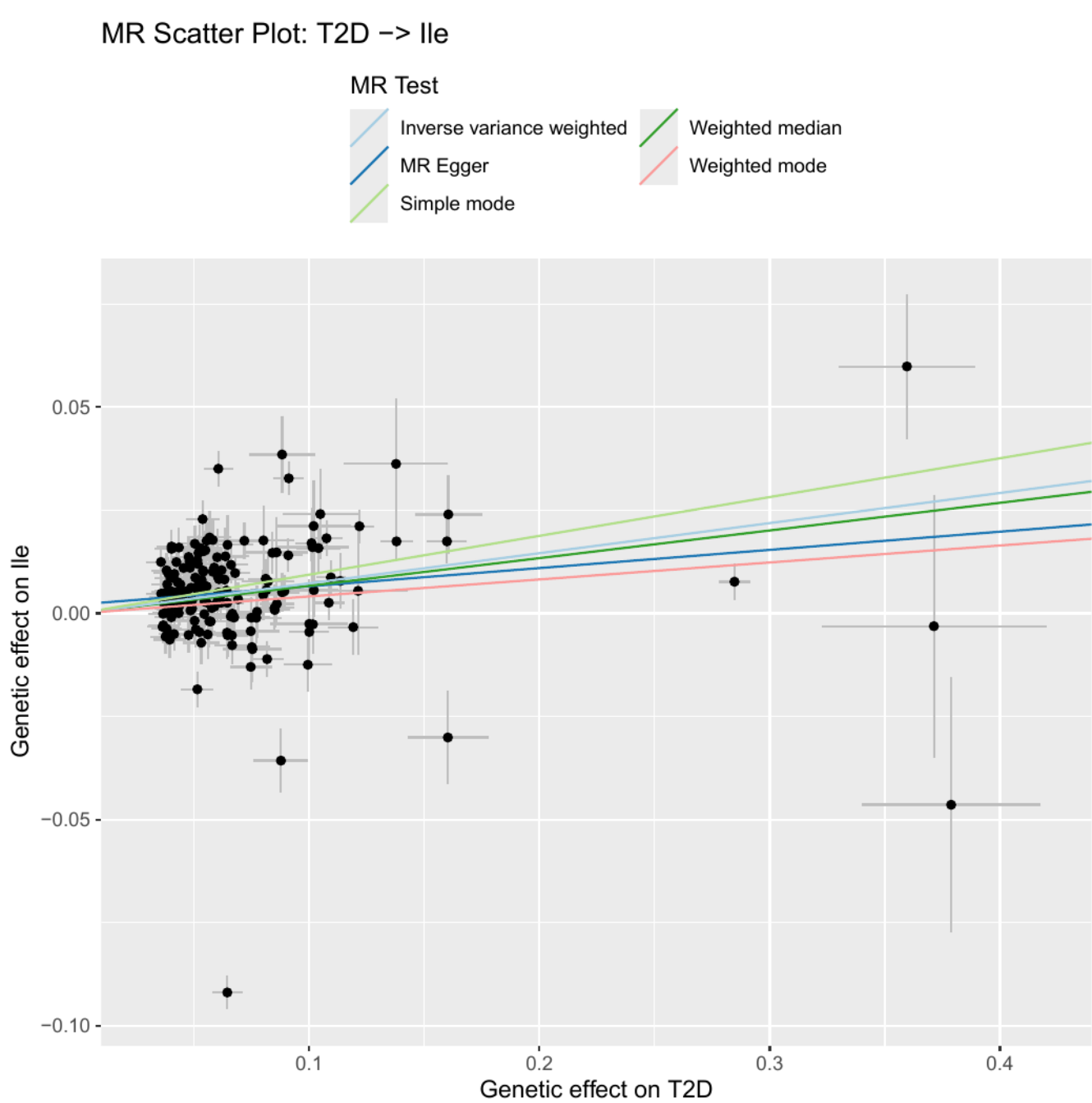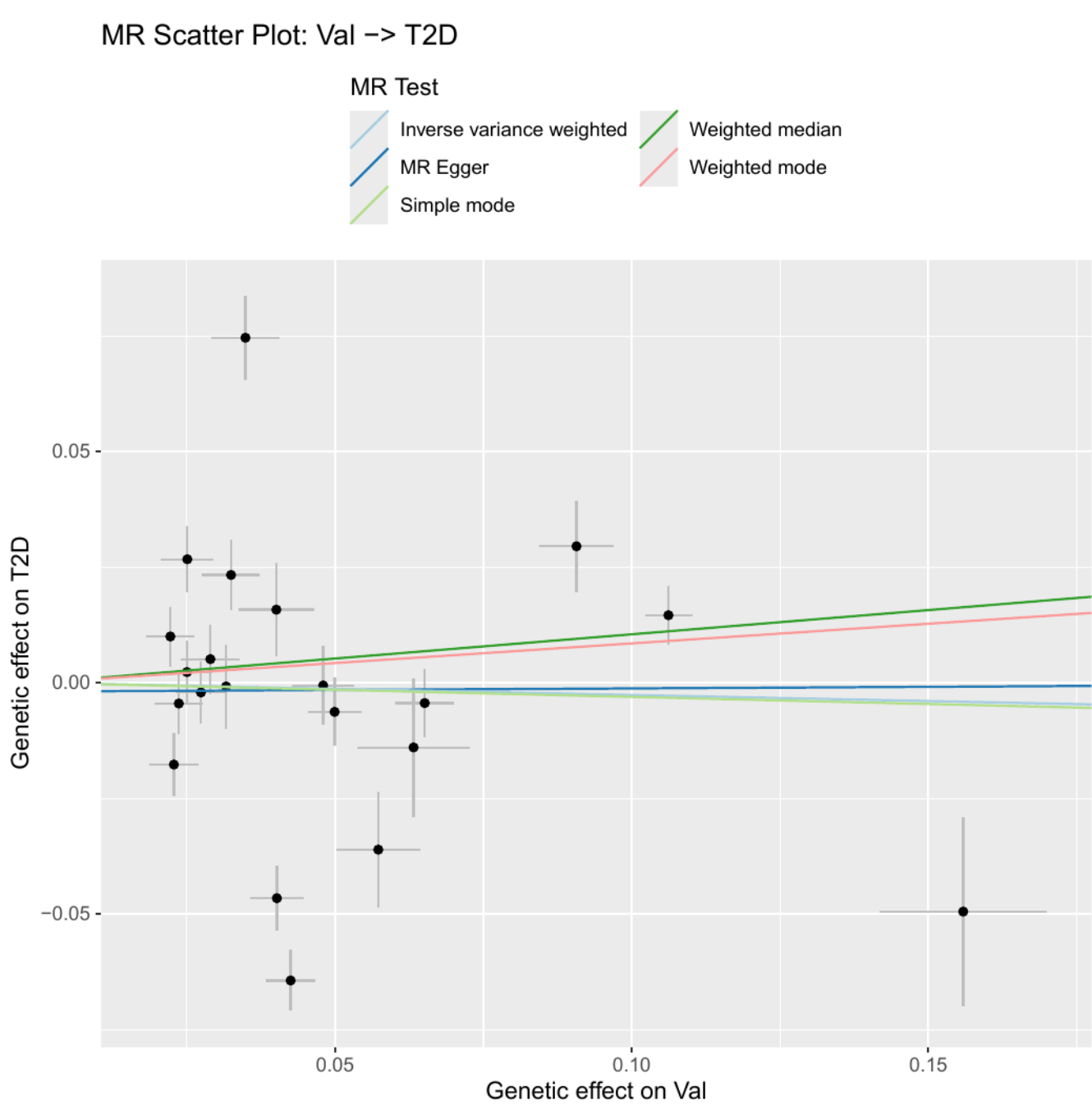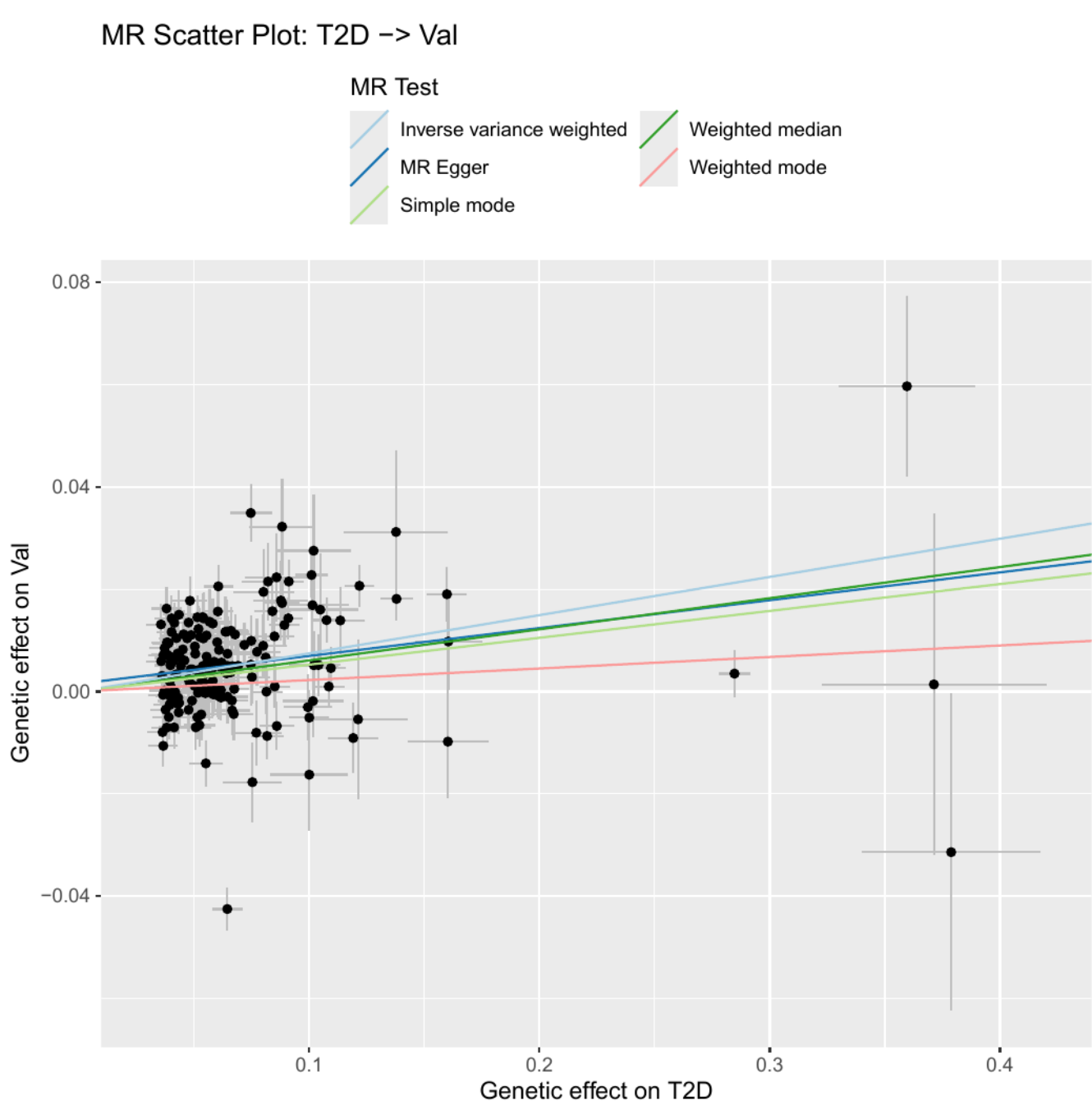

Supplement: Supplementary file 4 — Supplementary Material 4. [file 12920_2025_2232_MOESM4_ESM.pdf]

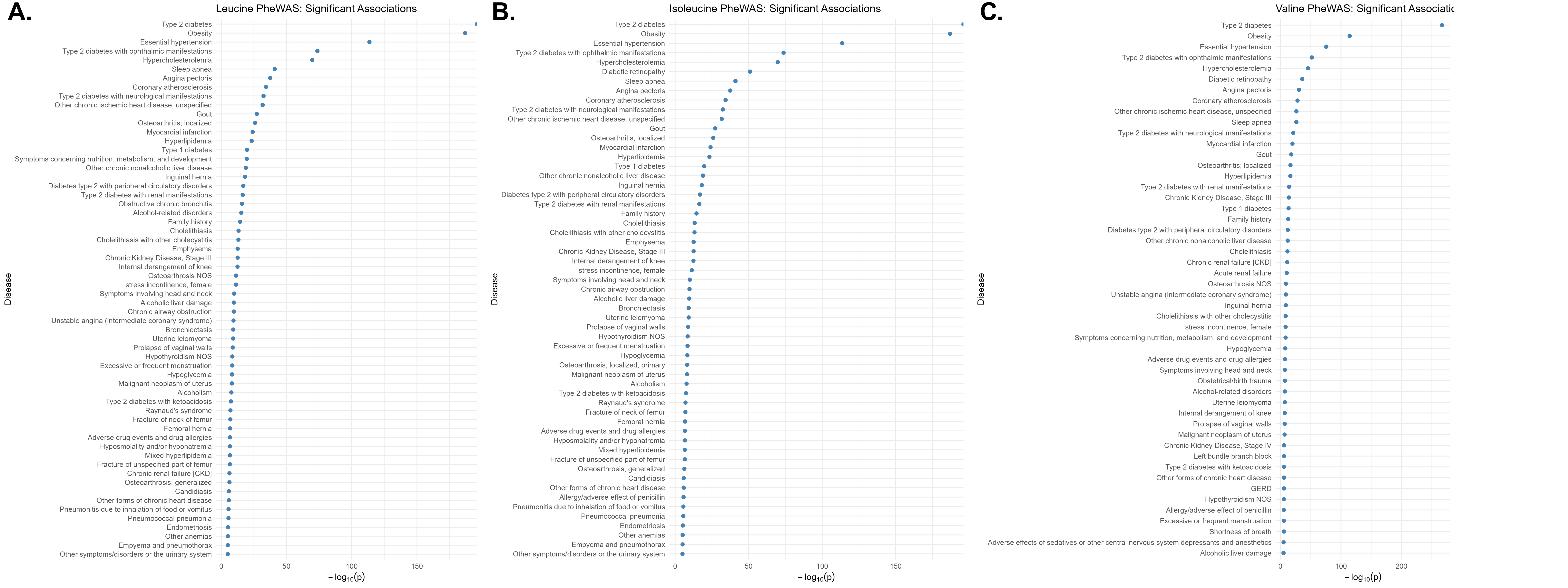

Supplement: Supplementary file 12 — Supplementary Material 12. [file 12920_2025_2232_MOESM12_ESM.png]
