# Supplementary material for: Assessment of genetic and metabolite associations of branched chain amino acids with metabolic disease in the UK Biobank using Mendelian randomization
Source: BMC Med Genomics. 2025 Oct 16;18:163. doi: 10.1186/s12920-025-02232-2 (PMC12532399; doi:10.1186/s12920-025-02232-2)
Supplement: Supplementary file 5 — Supplementary Material 5. [file 12920_2025_2232_MOESM5_ESM.pdf]

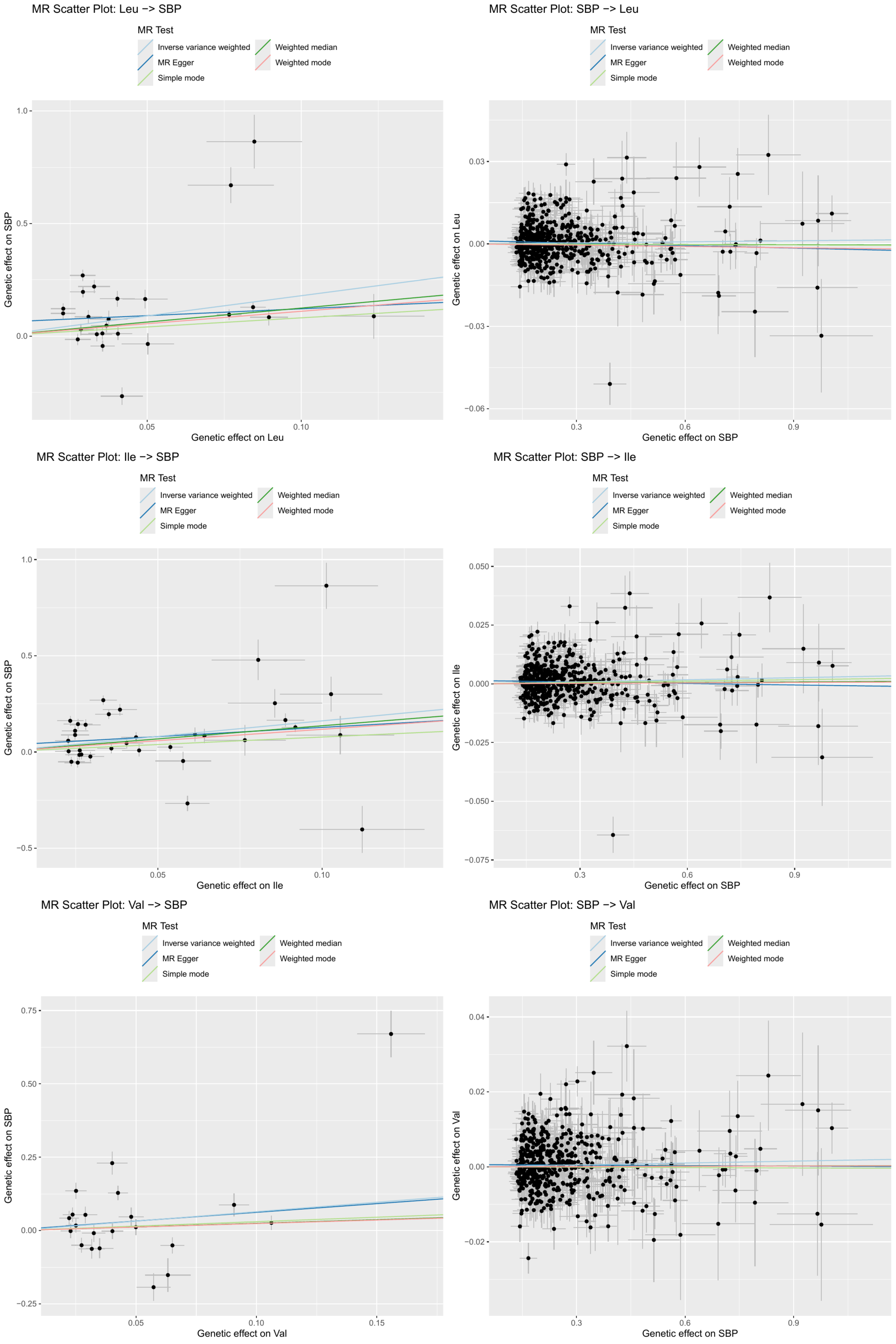
MR Scatter Plot: Leu −> SBP
MR Test
Inverse variance weighted
MR Egger
Simple mode
Weighted median
Weighted mode
Genetic effect on SBP
Genetic effect on Leu
1.0
0.5
0.0
0.05
0.10
MR Scatter Plot: SBP −> Leu
MR Test
Inverse variance weighted
MR Egger
Simple mode
Weighted median
Weighted mode
Genetic effect on Leu
Genetic effect on SBP
0.03
0.00
−0.03
−0.06
0.3
0.6
0.9
MR Scatter Plot: Ile −> SBP
MR Test
Inverse variance weighted
MR Egger
Simple mode
Weighted median
Weighted mode
Genetic effect on SBP
Genetic effect on Ile
1.0
0.5
0.0
−0.5
0.05
0.10
MR Scatter Plot: SBP −> Ile
MR Test
Inverse variance weighted
MR Egger
Simple mode
Weighted median
Weighted mode
Genetic effect on Ile
Genetic effect on SBP
0.050
0.025
0.000
−0.025
−0.050
−0.075
0.3
0.6
0.9
MR Scatter Plot: Val −> SBP
MR Test
Inverse variance weighted
MR Egger
Simple mode
Weighted median
Weighted mode
Genetic effect on SBP
Genetic effect on Val
0.75
0.50
0.25
0.00
−0.25
0.05
0.10
0.15
MR Scatter Plot: SBP −> Val
MR Test
Inverse variance weighted
MR Egger
Simple mode
Weighted median
Weighted mode
Genetic effect on Val
Genetic effect on SBP
0.04
0.02
0.00
−0.02
0.3
0.6
0.9
