# Supplementary material for: Assessment of genetic and metabolite associations of branched chain amino acids with metabolic disease in the UK Biobank using Mendelian randomization
Source: BMC Med Genomics. 2025 Oct 16;18:163. doi: 10.1186/s12920-025-02232-2 (PMC12532399; doi:10.1186/s12920-025-02232-2)
Supplement: Supplementary file 6 — Supplementary Material 6. [file 12920_2025_2232_MOESM6_ESM.pdf]

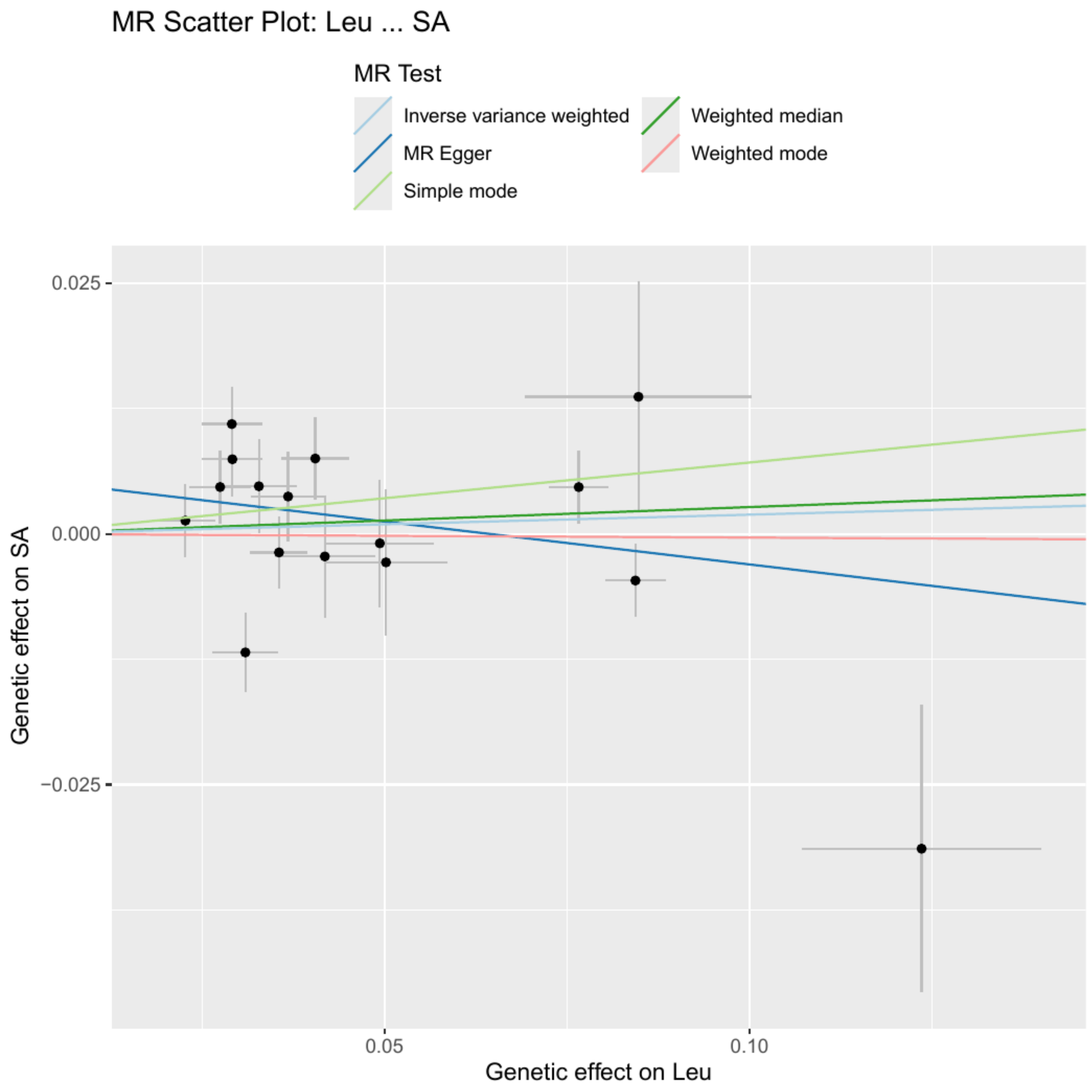
MR Scatter Plot: Leu ... SA
MR Test
Inverse variance weighted
MR Egger
Simple mode
Weighted median
Weighted mode
0.025
0.000
−0.025
0.05
0.10
Genetic effect on SA
Genetic effect on Leu

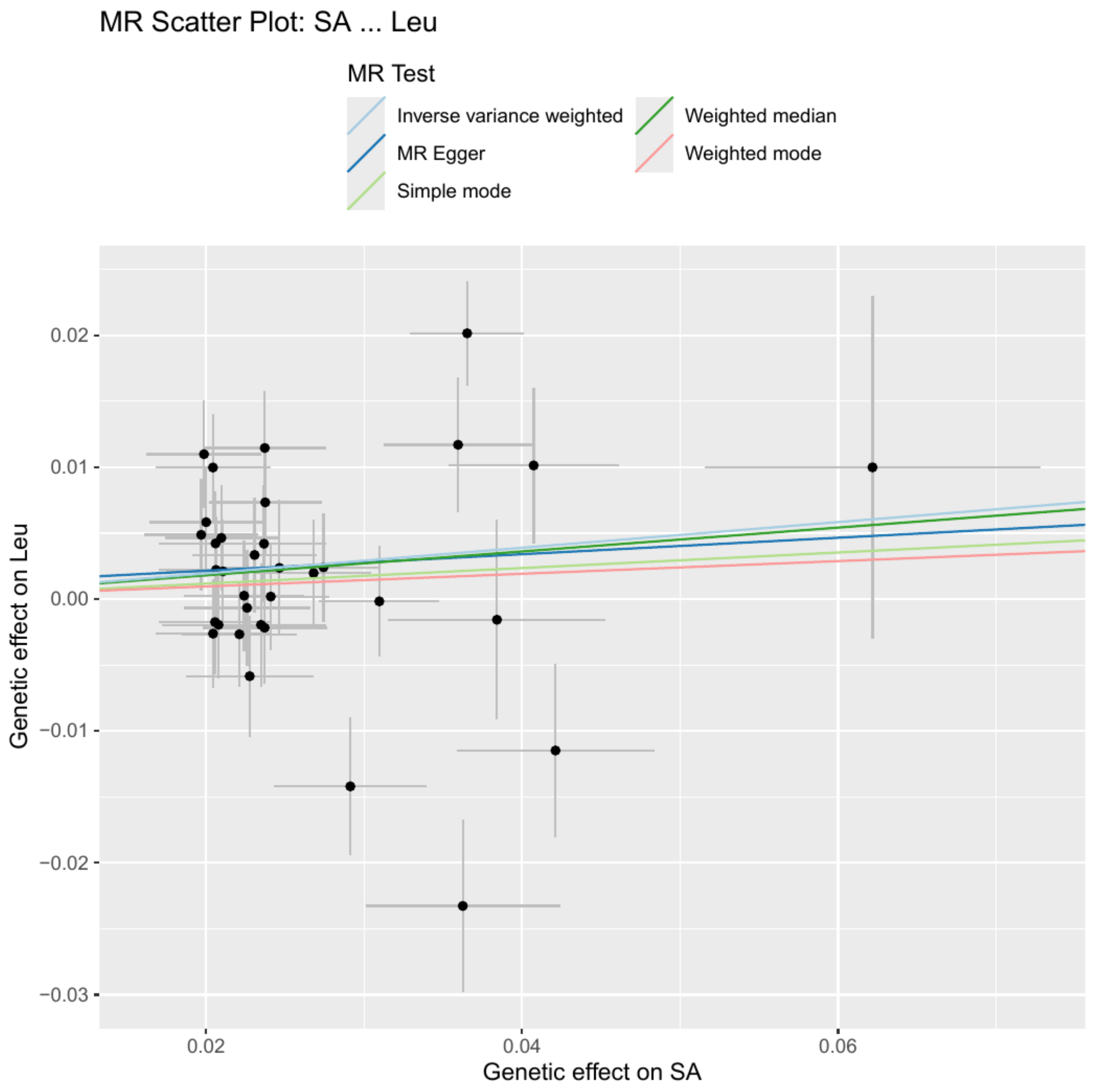
MR Scatter Plot: SA ... Leu
MR Test
Inverse variance weighted
MR Egger
Simple mode
Weighted median
Weighted mode
0.02
0.01
0.00
−0.01
−0.02
−0.03
0.02
0.04
0.06
Genetic effect on Leu
Genetic effect on SA

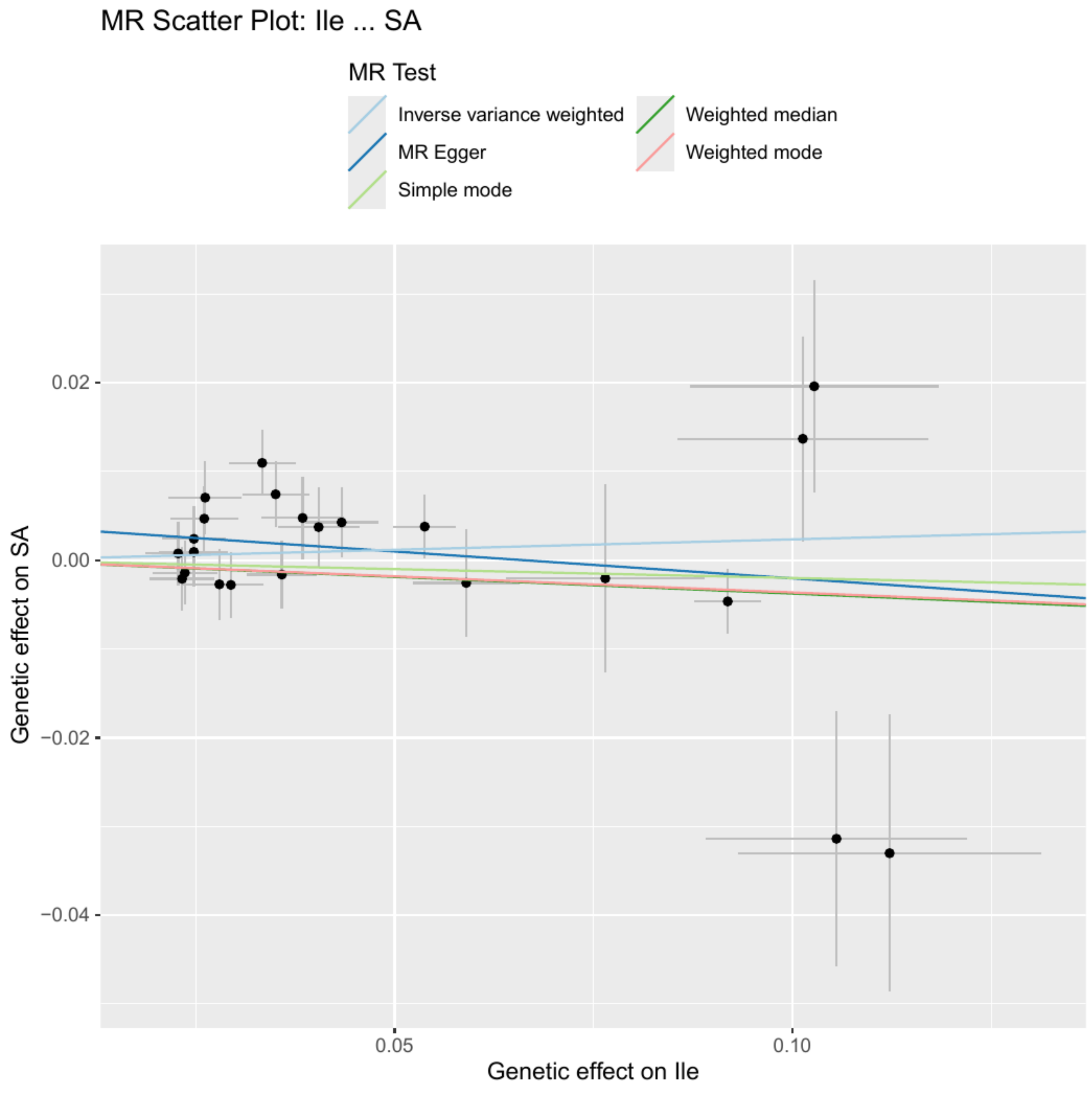
MR Scatter Plot: Ile ... SA
MR Test
Inverse variance weighted
MR Egger
Simple mode
Weighted median
Weighted mode
0.02
0.00
−0.02
−0.04
0.05
0.10
Genetic effect on SA
Genetic effect on Ile

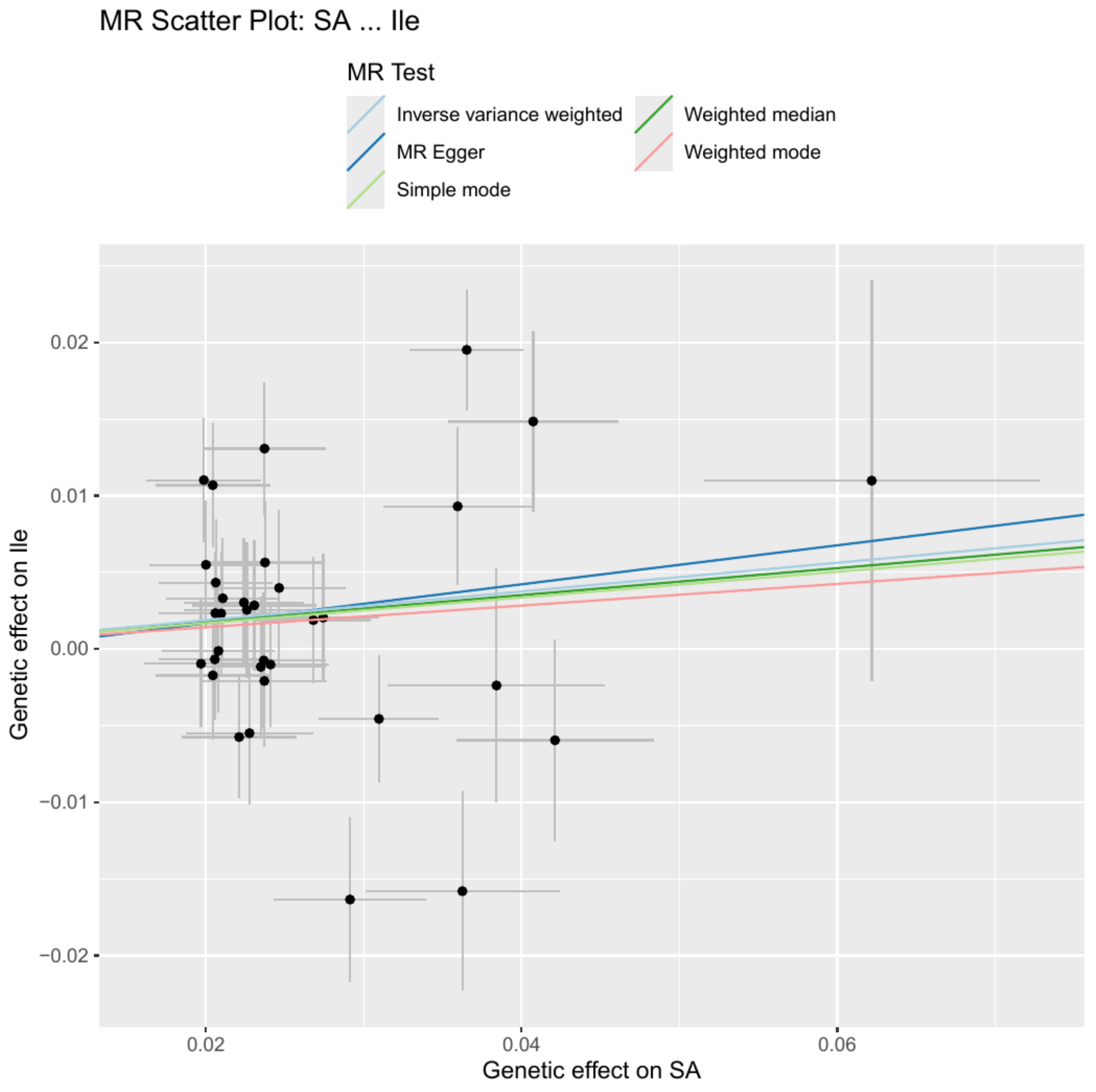
MR Scatter Plot: SA ... Ile
MR Test
Inverse variance weighted
MR Egger
Simple mode
Weighted median
Weighted mode
0.02
0.01
0.00
−0.01
−0.02
0.02
0.04
0.06
Genetic effect on Ile
Genetic effect on SA

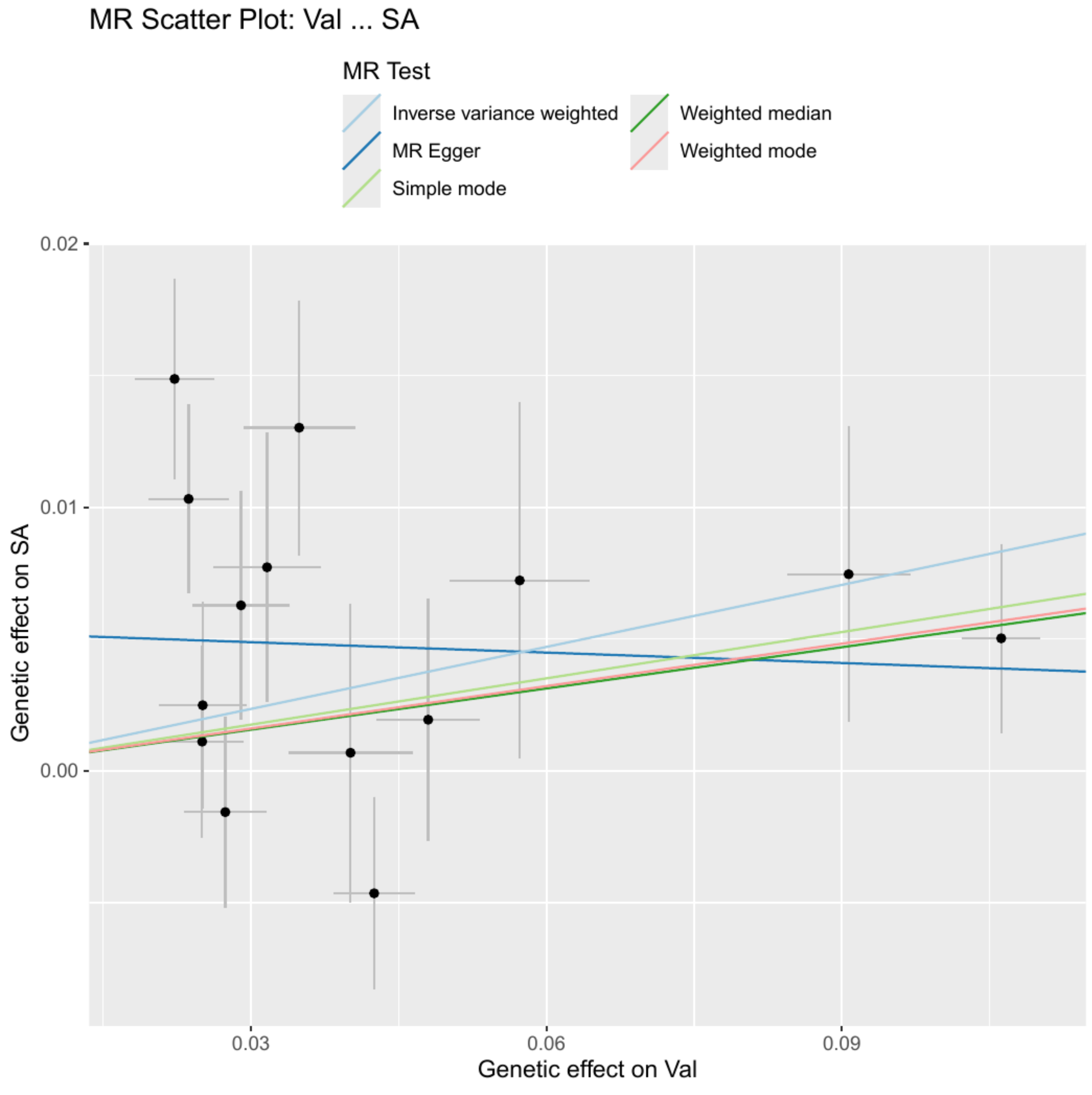
MR Scatter Plot: Val ... SA
MR Test
Inverse variance weighted
MR Egger
Simple mode
Weighted median
Weighted mode
0.02
0.01
0.00
0.03
0.06
0.09
Genetic effect on SA
Genetic effect on Val

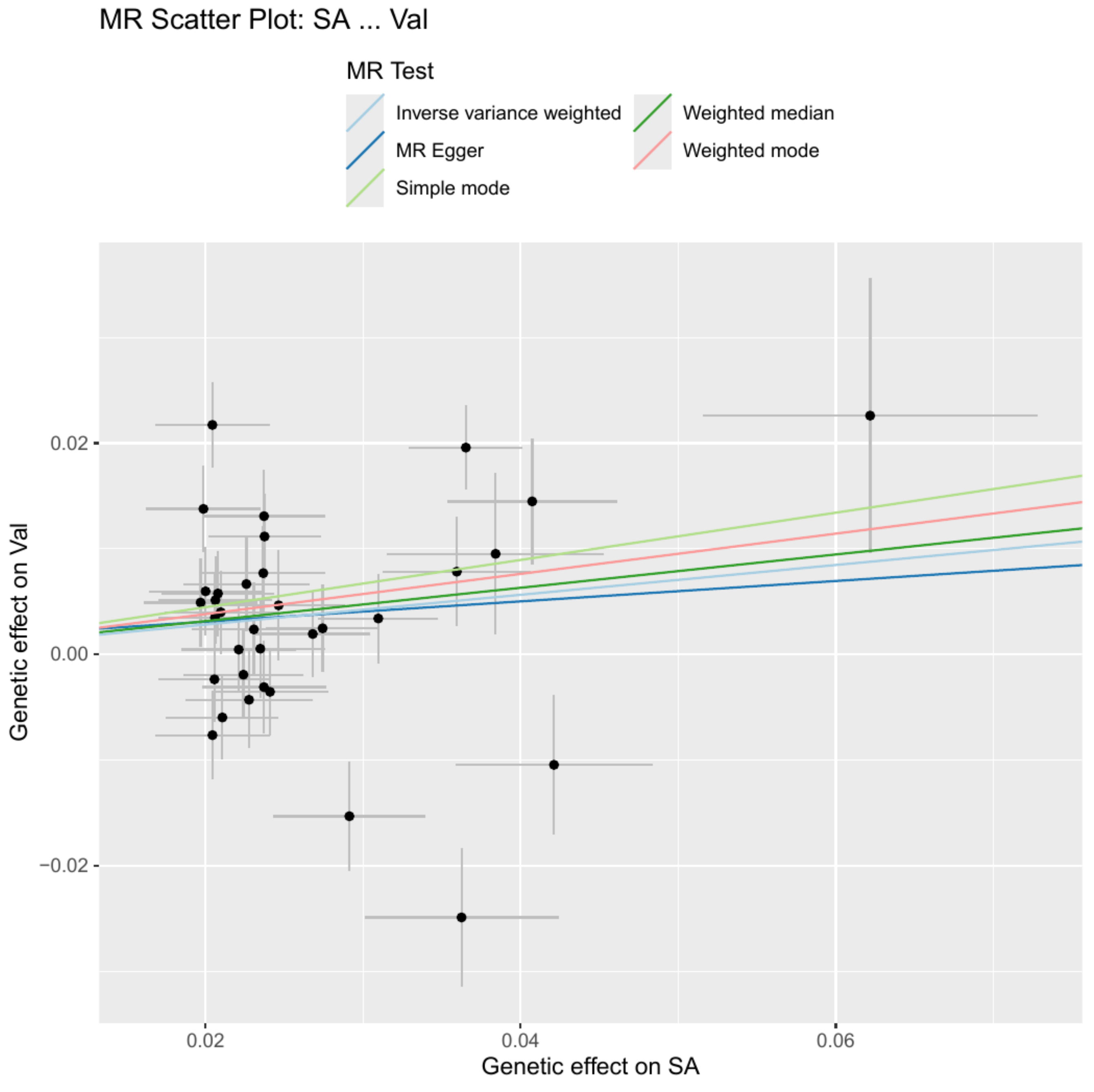
MR Scatter Plot: SA ... Val
MR Test
Inverse variance weighted
MR Egger
Simple mode
Weighted median
Weighted mode
0.02
0.00
−0.02
0.02
0.04
0.06
Genetic effect on Val
Genetic effect on SA
