# Supplementary material for: Assessment of genetic and metabolite associations of branched chain amino acids with metabolic disease in the UK Biobank using Mendelian randomization
Source: BMC Med Genomics. 2025 Oct 16;18:163. doi: 10.1186/s12920-025-02232-2 (PMC12532399; doi:10.1186/s12920-025-02232-2)

MR Scatter Plot: Leu -> BMI

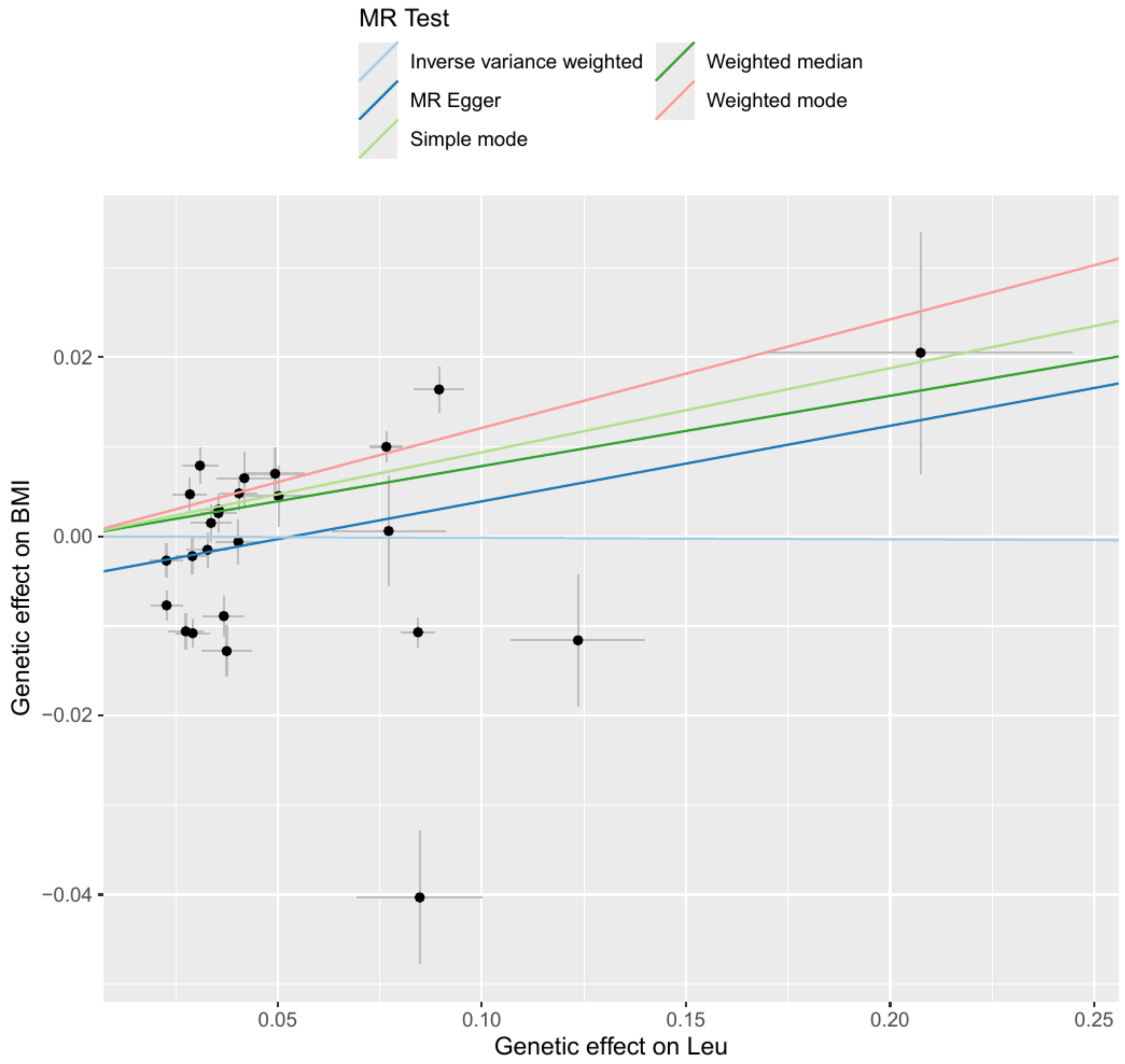

MR Scatter Plot: BMI -> Leu

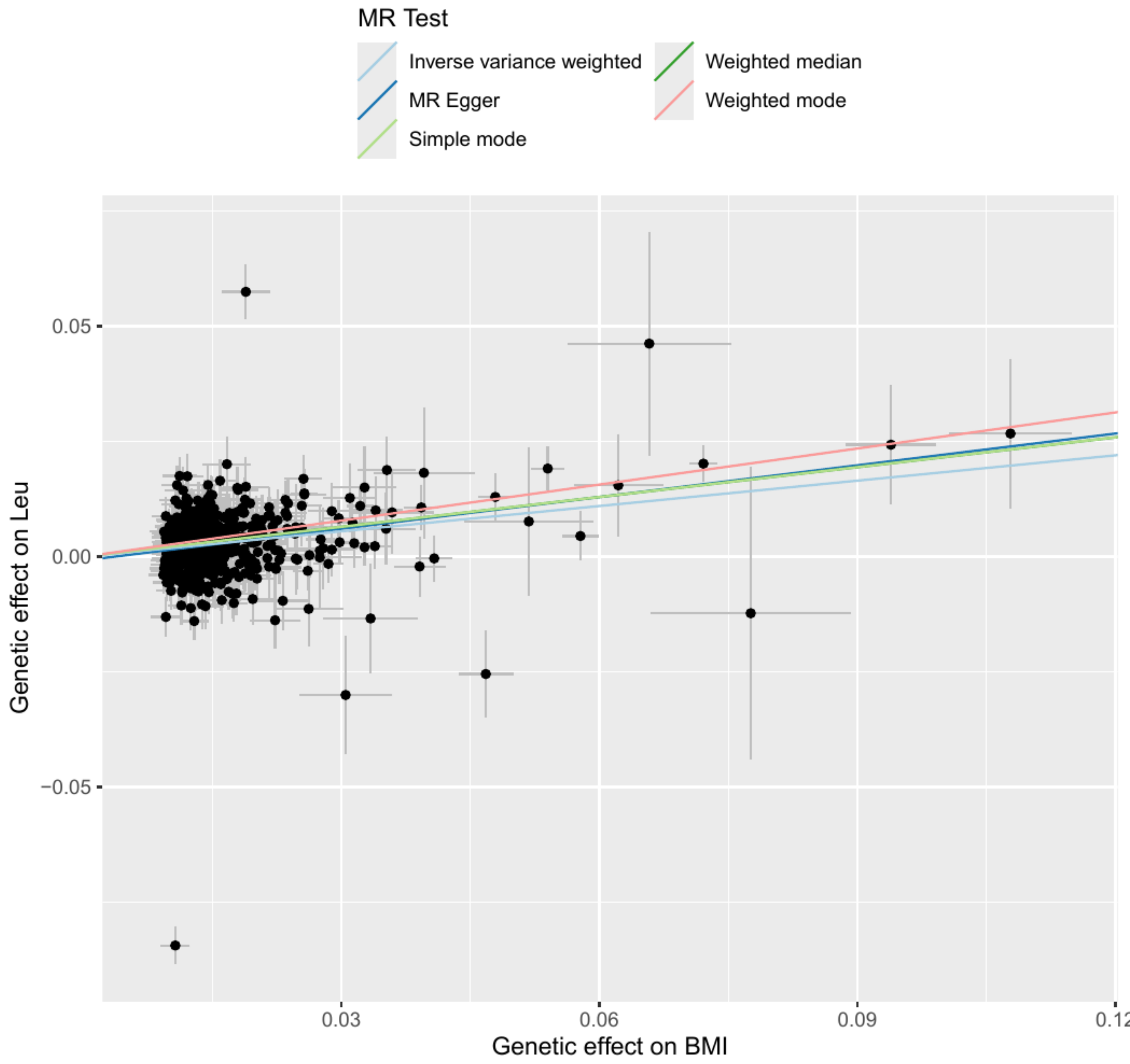

MR Scatter Plot: Ile -> BMI

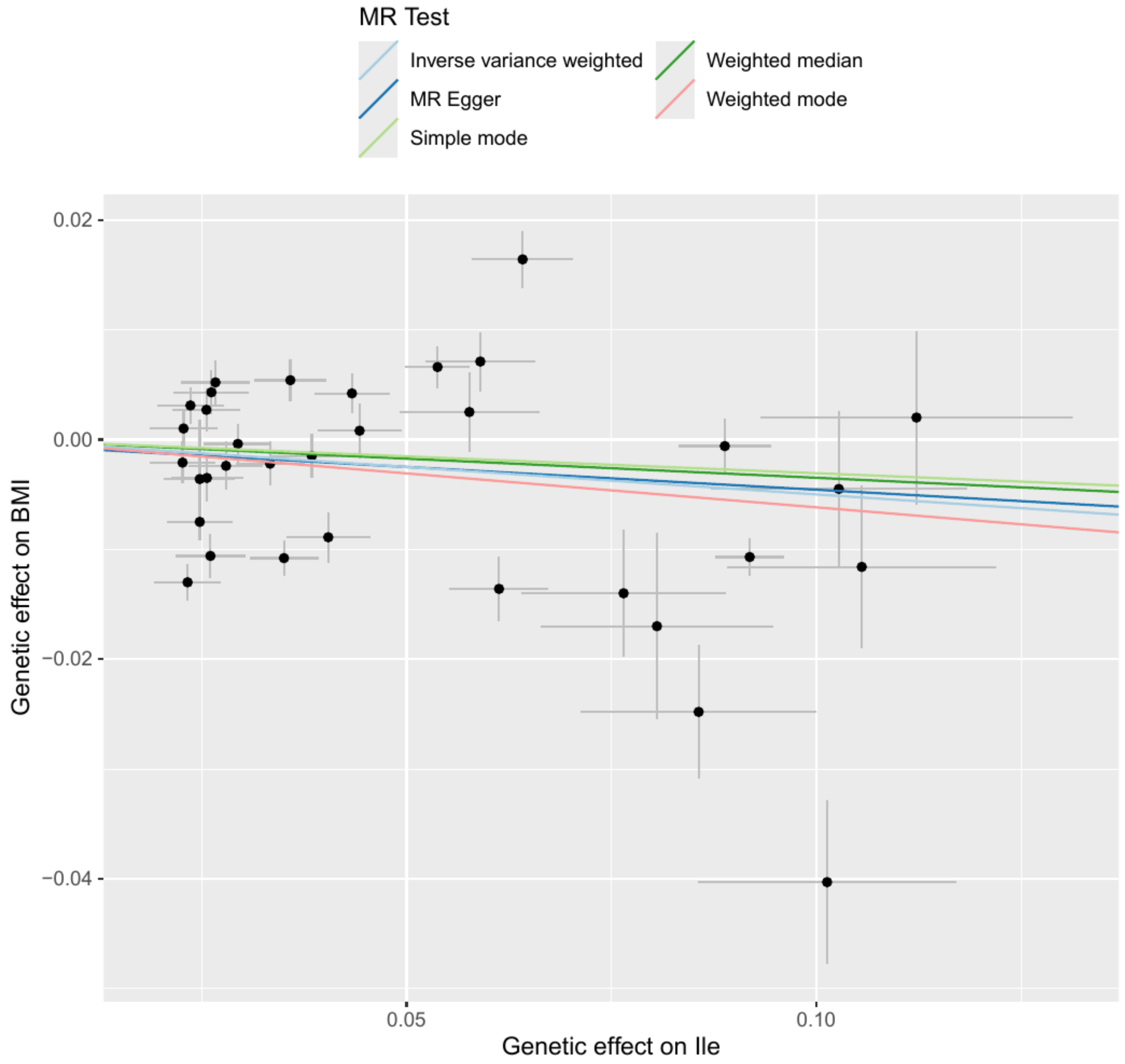

MR Scatter Plot: BMI -> Ile

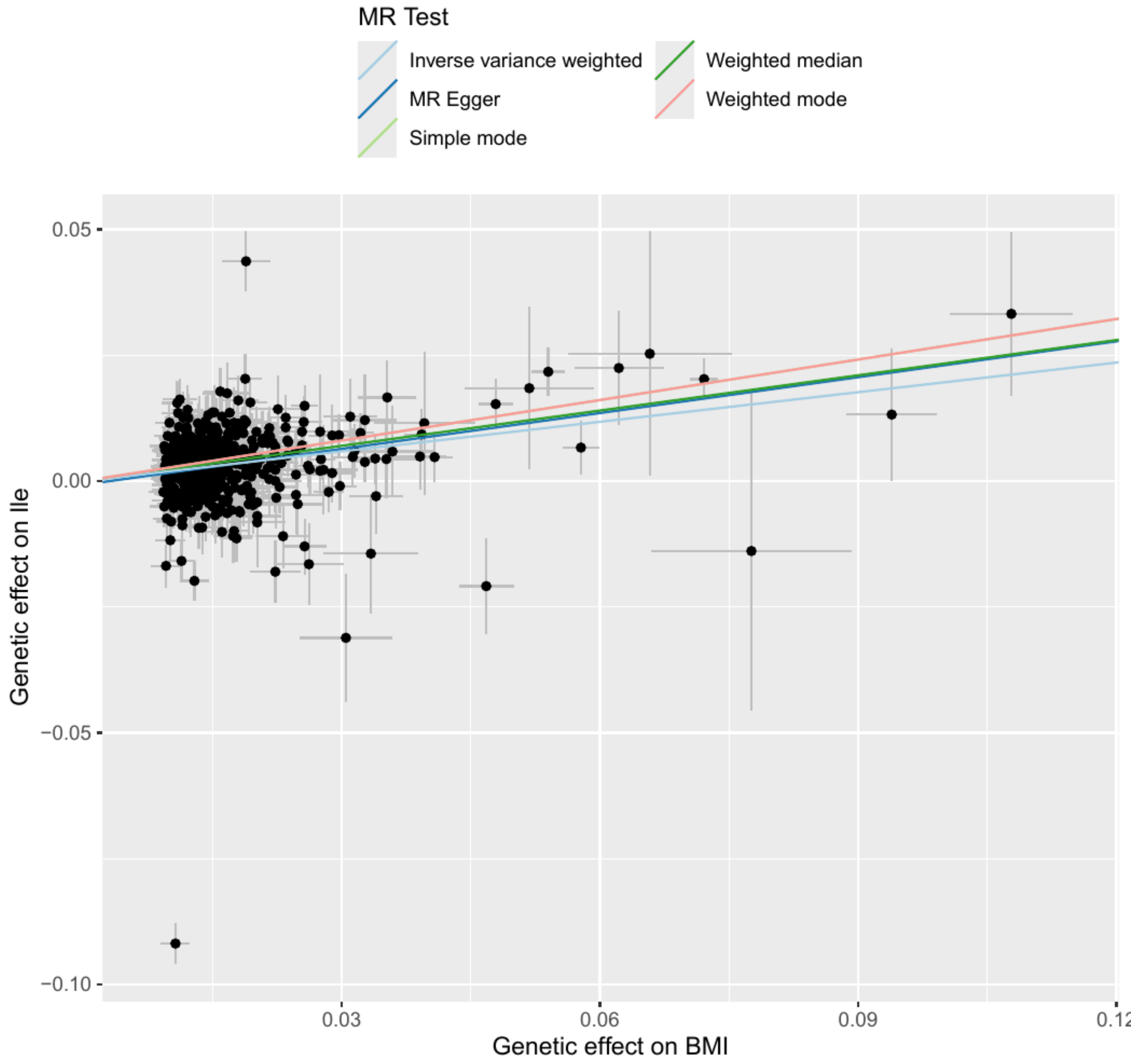

MR Scatter Plot: Val -> BMI

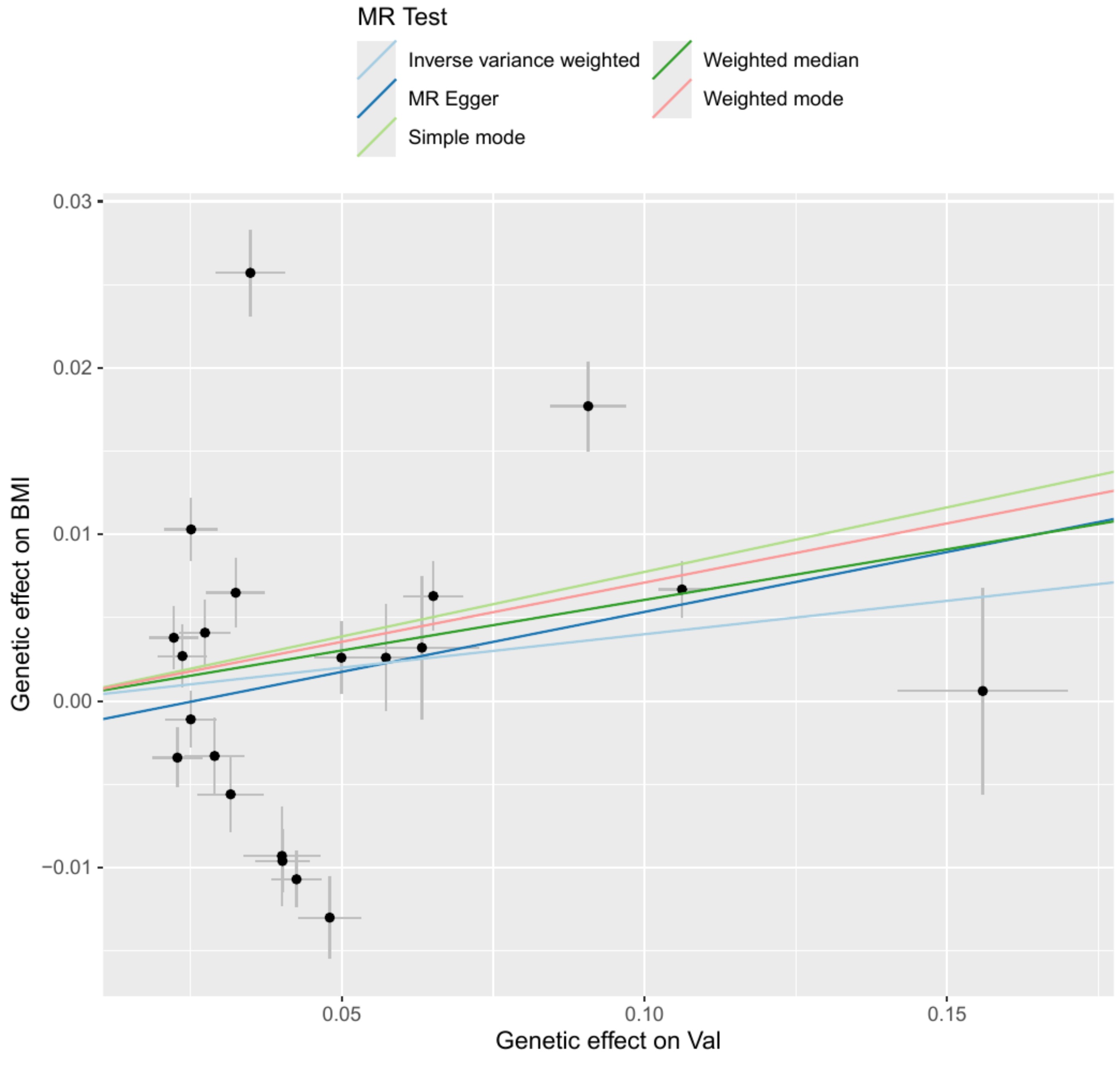

MR Scatter Plot: BMI -> Val

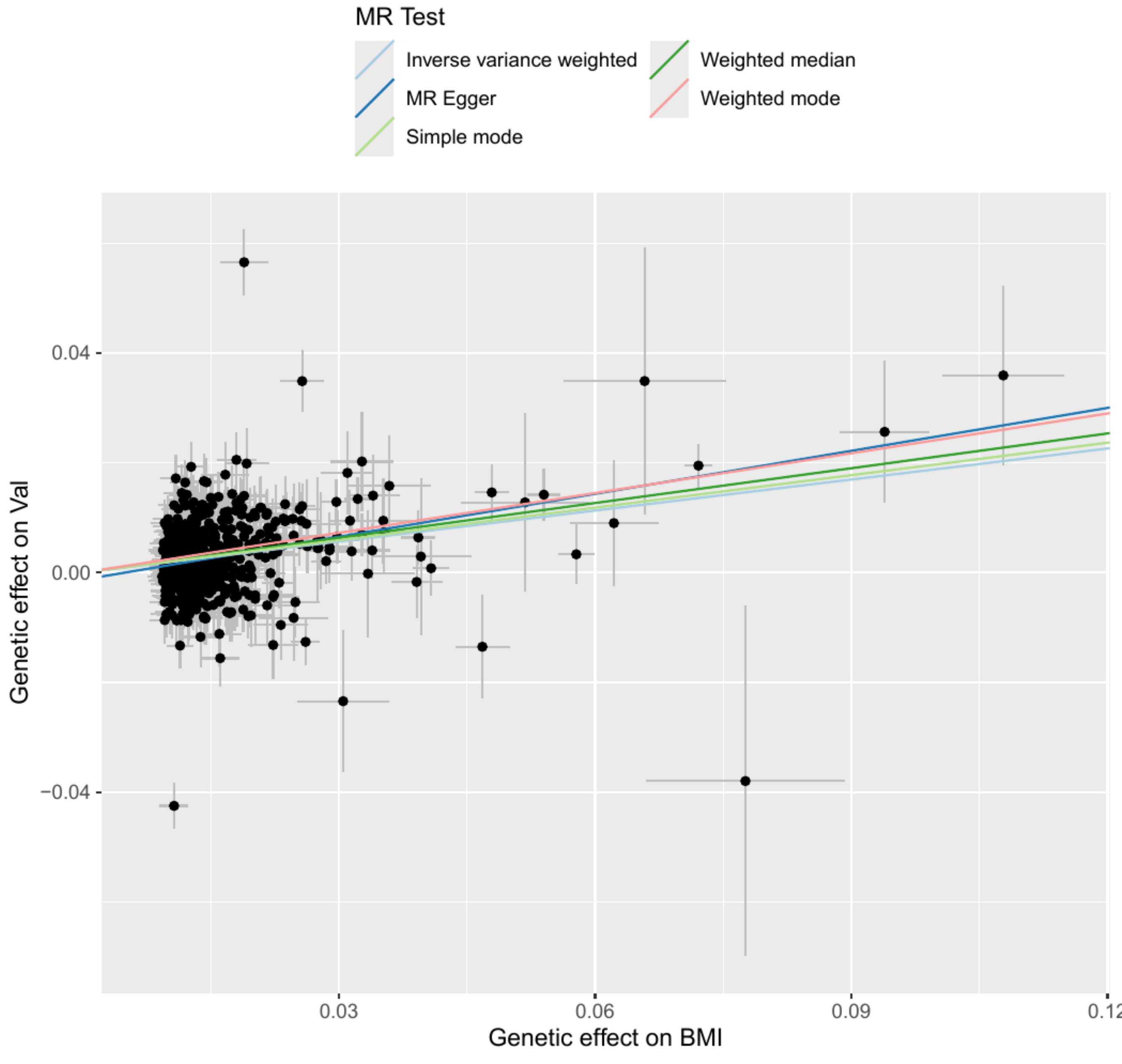

Supplement: Supplementary file 7 — Supplementary Material 7. [file 12920_2025_2232_MOESM7_ESM.pdf]
